# Taxonomic Revision and Molecular Phylogeny of the Genus *Morpheis* Hübner, [1820] (Lepidoptera: Cossidae)

**DOI:** 10.3390/insects17050458

**Published:** 2026-04-27

**Authors:** Artem E. Naydenov, Roman V. Yakovlev, Galina N. Shapoval, Fernando C. Penco, Anna E. Romanovich, Nazar A. Shapoval

**Affiliations:** 1 Institute of Biology and Biotechnology, Altai State University, Lenina Pr. 61, 656049 Barnaul, Russia; 2 X-BIO Institute, University of Tyumen, Volodarskogo St. 6, 625003 Tyumen, Russia; yakovlev_asu@mail.ru; 3 Institute of Biology, Tomsk State University, Lenina Pr. 36, 634050 Tomsk, Russia; 4 International Research Centre, Western Caspian University, 31 Istiglaliyyat Street, AZ1001 Baku, Azerbaijan; 5 Department of Karyosystematics, Zoological Institute, Russian Academy of Sciences, Universitetskaya emb. 1, 199034 St. Petersburg, Russia; galinakuftina@mail.ru; 6 Fundación de Historia Natural “Félix de Azara”, Departamento de Ciencias Naturales y Antropología, Universidad Maimónides, Hidalgo 775, Piso 7, Buenos Aires 1405BDB, Argentina; fernando_penco@hotmail.com; 7 Resource Center for Development of Molecular and Cellular Technologies, Saint-Petersburg State University, Universitetskaya emb. 7/9, 199034 St. Petersburg, Russia; aromanovich@gmail.com

**Keywords:** biodiversity, carpenter moths, neotropics, phylogeny, South America, taxonomy, Zeuzerinae

## Abstract

The genus *Morpheis* Hübner, [1820] (Lepidoptera: Cossidae) represents a taxonomically challenging group within Zeuzerinae, characterised by uniform, simply structured genitalia and significant intraspecific variability in external morphology, specifically in wing pattern, body colouration, and size. The taxonomic status and phylogenetic placement of many *Morpheis* taxa remain uncertain. Our study provides the first comprehensive taxonomic revision of *Morpheis*, based on detailed morphological analysis, examination of type specimens, assessment of distribution records, and molecular data. As a result, here we provide the first complete taxonomic list with updated diagnoses and distribution maps, based on 1247 specimen records and 191 publicly available observations. We describe the female genitalia of *Morpheis* for the first time and provide illustrations of imagoes for all currently recognised species, as well as of available type specimens. Additionally, we describe the immature stages and summarise the trophic associations of known *Morpheis* species. Based on morphological and molecular data, we recognise 11 valid *Morpheis* species. We applied molecular-based species delimitation methods, which uncovered far more candidate species within the genus than are recognised by traditional taxonomy, suggesting overlooked cryptic diversity that morphology alone cannot detect. We conclude that the integrative approach used here provides a powerful tool, especially valuable for understudied Zeuzerinae groups with high intraspecific variability in traditional characters.

## Simple Summary

The genus *Morpheis* is a group of carpenter moths within the subfamily Zeuzerinae, distributed across the New World, particularly in the Neotropical region. Despite their markedly different habitus, large size, and relatively frequent records in nature, representatives of this group have been poorly studied due to a lack of clearly defined diagnostic features and limited distribution data. This study presents a comprehensive revision of the genus, using DNA barcoding and morphological data. For the first time, we provide an updated taxonomic checklist, with revised diagnoses, distribution maps, and images of the adult moths and genitalia of all species in the genus. We conducted phylogenetic analyses using a 658 bp fragment of the *COI* mitochondrial gene, along with molecular species delimitation, including all but one traditionally recognised *Morpheis* taxa to delimit species boundaries and clarify their phylogenetic positions. We currently recognise 11 valid *Morpheis* species; however, molecular data suggest that species richness could be substantially higher due to overlooked cryptic diversity. Thus, these findings serve as a starting point for further integrative taxonomic studies on *Morpheis* diversity and phylogenetic relationships.

## 1. Introduction

The genus *Morpheis* Hübner, [1820] (Lepidoptera: Cossidae, Zeuzerinae) comprises relatively large carpenter moths distributed in the New World, primarily in the Neotropical region. Its range spans from the Mojave and Sonoran deserts in the north to the Monte Desert of Argentina in the south, excluding the high Andes and the West Indies. *Morpheis* belongs to Zeuzerinae, a widely distributed subfamily of Cossidae currently comprising 55 genera and 433 species [1]. Jacob Hübner established the genus for two species: *Phalaena* (*Hepialus*) *scalaris* Fabricius, 1775, and *Sphinx pyracmon* Cramer, (1780)] [2,3]. Subsequent designation by Roepke [4] fixed *S. pyracmon* as the type species of *Morpheis*, while *P. scalaris* became the type species of the genus *Azygophleps* Hampson, 1892 [5], which is widely distributed in the Old World, predominantly in the Paleotropics.

Houlbert [6] grouped most of the large-sized Neotropical Zeuzerinae under the subgenus *Neocossus* Houlbert, 1916, of the genus *Xyleutes* Hübner, [1820]. He designated *Endoxyla strigillata* Felder, 1874 (described from La Plata, Argentina) [7], as the type species of *Neocossus*, and included *X. cognatus* (Walker, 1856) [8], *X. melanoleuca* (Burmeister, 1878) [9], *X. palmarum* (Herrich-Schäffer, [1853]) [10], *X. pyracmon*, as well as two species described by himself (*X. mexicanus* Houlbert, 1916 and *X. oberthueri* Houlbert, 1916) in *Neocossus.* He assigned *X. xylotriba* (Herrich-Schäffer, [1853]) to a distinct subgenus *Strigocossus* Houlbert, 1916 pointing to its morphological similarity to the African taxon *X. crassa* (Drury, 1782). Houlbert proposed an Australian-Gondwanan origin for American *Xyleutes*, suggesting its dispersal to South America in two branches: the *xylotriba* and the *palmarum/strigillata* groups, followed by further expansion northward to Central America after the formation of the Isthmus of Panama.

Apparently unaware of Houlbert’s publication, A. J. Turner [11] established a new monotypic genus, *Xylotrypa* Turner, 1918, for *X. strigillata*.

Further significant taxonomic rearrangements appear in Seitz’s iconographies [12,13]. In particular, Gaede [12] corrected the type locality (initially listed as Australia) of *Phragmataecia impedita* Wallengren, 1860 [14] to South America and synonymised it with *X. strigillata.* Dyar [13] included in the genus *Xyleutes* the following species: *X. xylotriba*, *X. pyracmon*, *X. lelex* (Dognin, 1891) [15], *X. strigillata*, *X. melanoleuca*, *X. comisteon* (Schaus, 1911) [16], *X. cognata*, and described *X. discreta* Dyar, 1940. He also synonymised *Duomitus pyracmonides* Schaus, 1901 [17], *Zeuzera putrida* Percheron, 1838 [18], *Cossus palmarum* Herrich-Schäffer, [1853], and *Zeuzera fracta* Walker, 1856 with *X. pyracmon*; and *Duomitus mathani* Schaus, 1901 [17], *X. oberthueri*, and *X. mexicanus* with *X. cognata*. Additionally, Dyar assigned to this genus the species *X. strigifera* Dyar, 1910, *X. ramosa* (Schaus, 1892), *X. desdemona* Dyar, 1940, *X. masoni* (Schaus, 1894), and *X. albogrisea* Dognin, 1916, all of which were later reassigned to other genera such as *Brypoctia* Schoorl, 1990, *Carohamilia* Dyar, 1940, and *Psychonoctua* Grote, [1866] [19,20,21,22].

Donahue [23] revised the genus *Morpheis*, synonymizing *Neocossus* and *Xylotrypa*, and described the new species *M. clenchi* Donahue, 1980, from Arizona, USA. He assigned 12 species to *Morpheis*: *M. xylotribus*, *M. pyracmon*, *M. discretus*, *M. comisteus*, *M. lelex*, *M. strigillatus*, *M. impeditus*, *M. melanoleucus*, *M. votani* (Schaus, 1934) [24], *M. cognatus*, *M. mathani*, and *M. clenchi*. Unlike Dyar, Donahue considered *M. mathani* a valid species and synonymised *X. oberthueri* and *X. cognatus* Bryk, 1953 [25] with it. He distinguished the *cognatus* group within *Morpheis*, which includes *M. cognatus*, *M. mathani*, and *M. clenchi*, highlighting their diagnostic features and providing identification keys.

In 1991, J. W. Schoorl [19] published a monograph providing a detailed morphological description of *Morpheis* and a phylogeny of carpenter moths based primarily on external morphology (to a greater extent, on the analysis of the thorax sclerites, head appendages, and wing venation). He included 11 species in the genus: *M. melanoleucus*, *M. cognatus*, *M. mathani*, *M. strigillatus*, *M. pyracmon*, *M. fracta*, *M. putridus, M. xylotribus*, *M. votani*, *M. lelex*, and *M. clenchi*. According to Schoorl’s system, most *Morpheis* species remained morphologically indistinguishable, except for *M. melanoleucus,* which formed a distinct branch on the phylogenetic tree. Schoorl acknowledged that morphological characters alone are insufficient for reconstructing a reliable phylogeny, highlighting the high interspecific similarity among some species and the considerable intraspecific variability within others.

Finally, in 2021, a new species, *Morpheis humboldti* Naydenov, Yakovlev & Penco, 2021, was described from the Amazon regions of Brazil and Peru [26]. Thus, based on recent studies [19,23,26], the genus *Morpheis* is currently estimated to comprise approximately 11 to 13 species.

Despite well-defined key morphological characters and existing redescriptions of the genus *Morpheis*, the species composition and taxonomic status of many taxa remain controversial and debated among researchers [19,23,26]. No comprehensive illustrated overview of *Morpheis* species, covering type specimens and key diagnostic features, currently exists, resulting in frequent species misidentifications and the lack of a complete taxonomic revision. The genus (like many Zeuzerinae groups) exhibits uniform, simply structured genitalia lacking reliable diagnostic features, alongside high intraspecific variability in wing pattern, colouration of the head, thorax, and abdomen, as well as in size.

Although many Cossidae, particularly Zeuzerinae, are economically important pests of trees and shrubs, their trophic relationships remain fragmentarily studied due to the cryptic, xylophagous lifestyle of the caterpillars. Published data on the trophic associations of *Morpheis* pertain only to *M. pyracmon* [27,28], recorded as a stem endophage of *Mimosa pigra* (Fabaceae) and considered a potential biocontrol agent against this invasive plant. The HOSTS database (NHM, London) [29] lists a broad range of host plants for *Morpheis*. For *M. pyracmon* in Brazil (species-level identification requires verification), these include Arecaceae (*Syagrus romanzoffiana*), Fabaceae (*Mimosa caesalpiniifolia*, *M. scabrella*, *Senna alata*, *Inga* sp., with trunk development noted), and Salicaceae (*Salix babylonica*, in branches and trunk). For *M. strigillata* (currently considered as *M. impeditus*), Neotropical records list *Salix* (Salicaceae) and *Citrus* (Rutaceae). For *M. xylotribus* in Brazil, host plants include *Citrus* (Rutaceae), *Mimosa sordida*, and *Inga* sp. (both Fabaceae). An iNaturalist observation [30] documents an *M. pyracmon* imago emerging from wood of *Senegalia polyphylla* (Fabaceae).

Thus, available data allow only tentative speculation regarding the trophic relationships of *Morpheis*. Data on the biology of its species require targeted taxonomic and ecological verification and currently provide insufficient grounds for reliable conclusions on food specialisation or the actual spectrum of host plants within the genus.

The rapid implementation of new molecular techniques and approaches over recent decades, such as DNA barcoding, has provided a powerful analytical tool for species identification and delimitation, unveiling cryptic diversity and revealing interspecific and deep-level relationships. Although it has certain limitations [31,32], DNA barcoding has efficiently resolved many longstanding taxonomic problems in Lepidoptera, including the family Cossidae [33,34,35,36,37,38].

Despite the broad and constantly growing use of DNA-based techniques in Lepidoptera taxonomy and molecular systematics, studies focused on *Morpheis* are virtually absent, and molecular data for the genus remain very limited. Thus, DNA barcodes of *M. pyracmon* and *Brypoctia ramosa* (erroneously attributed to *Morpheis*) were used to root the phylogeny of Indonesian *Zeuzera* Latreille, 1804 [39]; *M. xylotribus* and an unidentified *Morpheis* specimen were included in a phylogenetic analysis of selected Cossidae groups aimed at clarifying intergeneric relationships within the family [40]. In two broader studies, a *Morpheis* specimen was used as a representative of Cossoidea to infer higher-level phylogenetic relationships within Lepidoptera [41,42]. Consequently, no currently published molecular phylogenetic studies have been devoted exclusively to *Morpheis* itself. Although the Barcode of Life Database (BOLD) currently includes a substantial number of records assigned to *Morpheis* (https://portal.boldsystems.org/result?query=Morpheis[tax]) (accessed on 15 February 2026), they are limited to only the most common and widespread species and typically originate from large series of specimens collected at the same site. Moreover, many of these barcoded specimens are erroneously attributed to *Morpheis*, being actually members of other cossid genera.

The aim of the present study is to provide a comprehensive taxonomic review of the genus *Morpheis*, including illustrations of all available type specimens, adult habitus, and male genitalia of all species, as well as female genitalia where material is available. We clarify the taxonomic status of problematic taxa, synonymy, and diagnostic characters, and provide updated diagnoses for all species. We summarise species distribution and provide distribution maps based on verified museum specimens and selected georeferenced records. For the first time, we present a molecular phylogeny of the genus, including all species except *M. melanoleucus*, for which comparable genetic data are unavailable. Taken together, these results provide an essential basis for future revisions of Zeuzerinae and enhance species identification accuracy in museum collections and field studies.

## 2. Materials and Methods

### 2.1. Morphological Analysis and Mapping

We examined a total of 1247 specimens (including type specimens) of *Morpheis* species stored in 16 state/public museums and six private collections (see List of Abbreviations). Species identification was based on examination of type specimens, original descriptions and illustrations, taking type localities into account. To achieve a more precise delineation of species distributions, we analysed a subset of 191 observations of *Morpheis* from the iNaturalist project “New World Zeuzerinae Observations” [43], selecting exclusively those suitable for reliable species identification. Counts of examined specimens for each *Morpheis* species, including selected iNaturalist observations, are given in Supplementary Table S1.

Imagoes were photographed using Canon EOS 600D, 70D, and 6D Mark II cameras (Canon Inc., Tokyo, Japan) under controlled Lightbox illumination. Male and female genitalia were dissected and slide-mounted following standard procedures [44,45,46]. Preparations were stained with eosin and Evans Blue and preserved as permanent slides in Euparal mounting medium (Carl Roth GmbH + Co. KG, Karlsruhe, Germany). Genital preparations were examined with MBS 10 (USSR), Zeiss Stemi 2000 C (Carl Zeiss Microscopy GmbH, Jena, Germany), and Olympus SZX16 microscopes (Olympus Corporation, Tokyo, Japan) and photographed using Olympus DP74 (Olympus Corporation, Tokyo, Japan) and Canon EOS 70D cameras (Canon Inc., Tokyo, Japan). Images were processed edited and compiled using Adobe Photoshop CS6 13.0.1 software. The distribution maps were generated using the web-based tool SimpleMappr (https://www.simplemappr.net/) (accessed on 15 September 2025). Morphological terminology follows Kristensen [47]. The biogeographic regionalization of the world (specifically the Neotropical region) follows Morrone [48,49].

### 2.2. DNA Extraction, PCR, and Sequencing

One leg from each specimen was taken for DNA extraction. DNA was isolated using the DNeasy Blood and Tissue Kit (Qiagen, Inc. Valencia, CA, USA) following the manufacturer’s protocols and the CTAB-based method [50], with modifications [51,52,53]. Samples were processed in two laboratories: at the Department of Karyosystematics, Zoological Institute of the Russian Academy of Sciences (St. Petersburg, Russia) and at the DNA Lab, Natural History Museum, University of Oslo (Oslo, Norway), within the framework of the “Norwegian–Russian network for training the new generation of entomologists in DNA-based molecular methods (NoREnt)”.

The mitochondrial DNA barcode (a 658 bp fragment of the *COI* gene) was amplified using LCO1490/HCO2198 [54] and LepF/LepR primer pairs [55]. In cases where standard lepidopteran barcode primers failed to yield a sufficient product, full-length barcode fragments were amplified using the primer pair combinations LepF/MH-MR1 + MH-MF1/LepR and LCO1490/MH-MR1 + MH-MF1/HCO2198 [56].

PCR amplifications were performed under conditions previously described [57,58]. The success of PCR amplification was evaluated by electrophoresis of the products in 1% agarose gel. The purified PCR products were subjected to further sequencing. Sequencing of the double-stranded products was carried out at the Research Resource Center for Molecular and Cell Technologies (Scientific Park, St. Petersburg State University, St. Petersburg, Russia) within the framework of state assignment No. 125022803066-3. The obtained *COI* sequences were deposited in the publicity available BOLD project “MORSA” under accession numbers MORSA001-26—MORSA031-26.

### 2.3. Molecular Data Analysis and Phylogenetic Reconstructions

All sequences were checked for errors, edited and aligned using Geneious v.8.1.6 [59] and BioEdit v.7.0.3 [60] software. Primer sequences were cropped. A total of 31 specimens of the genus *Morpheis* were sequenced during this study. Additionally, 254 *COI* sequences of the genus available in BOLD were included in the phylogenetic analysis. This dataset excluded short sequences and records erroneously attributed to *Morpheis*. The list of the specimens used for the molecular analysis and their collection data are given in Supplementary Table S2. The barcode of the closely related genus *Brypoctia* (*B. punctifer* (Hampson, 1898)) was used as the outgroup to root the phylogeny. Thus, the final *COI* dataset (alignment length 658 bp) for the phylogenetic analysis included 286 sequences.

Phylogenetic analyses were performed using the Bayesian inference (BI) and Maximum Likelihood (ML) approaches. jModelTest v.2.1.7 [61] was used to find the best substitution model for the current data set. Bayesian analysis was performed in MrBayes v.3.2.7a software [62] under the nucleotide substitution model GTR + G + I. Parameters were estimated using two independent runs of 10 million generations each, with four simultaneous chains (one cold and three heated). Trees and parameters were sampled every 1000 generations. The first 25% of trees were discarded as burn-in prior to computing a consensus phylogeny and posterior probabilities. TRACER v.1.6 was used to check the stationarity and convergence of Bayesian analyses between runs [63]. The consensus trees were visualised using FigTree v.1.4.4 [64]. The ML analysis was performed using the IQ-TREE v.3.0.1 software [65] setting an automatic selection of models. Branch support was evaluated through 1000 replications of Ultrafast Bootstrap (UFB) [66] and Shimodaira-Hasegawa-like approximate likelihood ratio test (SH-aLRT) [67]. Genetic distances among *COI* barcodes were calculated in MEGA v.7.0.14 [68] and are given in Supplementary Tables S3 and S4. To test the taxonomic status of recovered clades, we used the recently developed method ASAP, Assemble Species by Automatic Partitioning [69], which proposes partitions of species hypotheses based on genetic distances calculated between DNA sequences.

## 3. Results

### 3.1. Phylogenetic Relationships Within the Genus Morpheis

#### 3.1.1. Maximum Likelihood (ML) and Bayesian Inference (BI) Analyses

**Figure 1:**
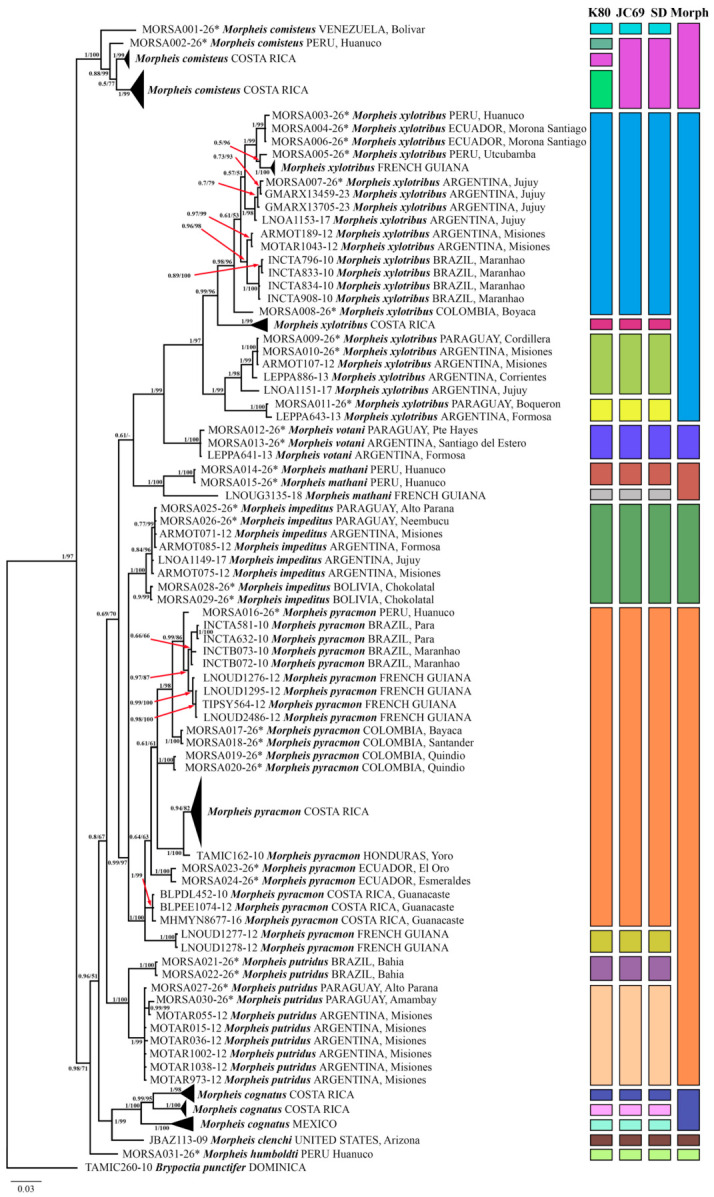
Phylogenetic relationships of *Morpheis* species inferred from *COI* barcodes. Asterisks (*) indicate specimens sequenced in this study. Numbers at branches indicate Bayesian posterior probabilities (before the slash) and ML ultrafast bootstrap support values (after the slash). Vertical bars on the right correspond to morphologically defined *Morpheis* species (Morph) and to species boundaries suggested by the ASAP method under the Kimura-2P (K80), Jukes–Cantor (JC69), and uncorrected p-distance (SD) models.

We performed maximum likelihood (ML) and Bayesian Inference (BI) analyses to reconstruct phylogenetic relationships. Both analyses recovered nearly identical *COI* tree topologies with largely consistent support values (Figure 1). For visual clarity, sequences from individuals of the same locality (or region) with similar barcodes were collapsed (the uncollapsed BI tree is shown in Supplementary Figure S1). BI and ML analyses recovered the genus *Morpheis* as a strongly supported monophyletic lineage. In general, low-level (species-level) phylogenetic relationships were well resolved and strongly supported across most nodes, whereas deeper-node (intergeneric) topology remained largely unresolved.

Samples morphologically assigned to *M. comisteus* formed a strongly supported basal lineage sister to the remaining *Morpheis*. In turn, this lineage split into four clades with moderate to high branch support: two comprising specimens from Costa Rica, one with a single *M. comisteus* specimen from Peru, and one with a single *M. comisteus* specimen from Venezuela. The next basal lineage recovered by BI and ML analyses comprised a specimen of the recently described species *M. humboldti* [26] from Peru. A further split, well-supported in BI (posterior probability (PP) >0.95), but unsupported in ML (UFB = 51), recovered a clade with a *M. clenchi* specimen as sister to three strongly supported clades of *M. cognatus* (two comprising specimens from Costa Rica and one comprising specimens from Mexico). After the divergence of *M. clenchi* and *M. cognatus*, phylogenetic analyses recovered the *M. putridus* lineage as sister to the rest of the species diversity. Within this lineage, two monophyletic groups with high branch support are recognised: the first comprising specimens geographically restricted to Brazil, and the second composed of specimens from Paraguay and Argentina. It should be noted that *M. putridus* is indistinguishable from its sibling species *M. pyracmon* in morphological characters, including wing pattern and male genitalia structure. However, our BI and ML analyses reveal a distinct phylogenetic placement of these two taxa: *M. pyracmon* appears to be more closely related not to *M. putridus*, as expected, but to several other *Morpheis* taxa. On the phylogenetic tree, *M. pyracmon* clusters as a sister clade to *M. impeditus* with high support (PP = 0.99, UFB = 97), rather than forming a clade with *M. putridus*, highlighting an existing incongruence between morphology and molecular phylogeny. The next deep-level branching was unsupported by either BI or ML analyses. It was sustained, however, by several highly supported branches of *M. mathani*, *M. votani*, and *M. xylotribus*. The *M. mathani* clade appeared with maximum support as a sister basal clade to (*M. votani* + *M. xylotribus*). It was divided into two groups: the first comprising *M. mathani* specimens from French Guiana, and the second including *M. mathani* specimens from Peru. Specimens of *M. votani* were recovered by phylogenetic analyses as a single, strongly supported (PP = 1, UFB = 100) monophyletic entity, sister to *M. xylotribus*. The last clade is composed of *M. xylotribus* specimens from various geographic localities. Two deep-branching lineages within this clade were recovered on the phylogenetic tree; however, these lineages are inconsistent with specimen geography. In turn, each clade is further subdivided into numerous subclades.

Intraspecific barcode divergence in *Morpheis* varied greatly depending on species, ranging from 0.3% (±0.2%) in *M. votani* to 9.4% (±1.1%) in *M. xylotribus* (Table S4). The lowest intraspecific divergence values were observed in species with small numbers of sequenced specimens collected at a single site or a few geographically proximate localities. In contrast, intensively barcoded species with broad geographic sampling showed markedly high intraspecific divergence, exceeding 9%. Interspecific differences within the genus ranged from 4.9% ± 0.8% (between *M. impeditus* and *M. humboldti*) to 14.3% ± 1.3% (between *M. cognatus* and *M. xylotribus*) (Table S3).

#### 3.1.2. Species Delimitation

The species delimitation analysis was based on DNA barcodes of 285 *Morpheis* specimens (without inclusion of an outgroup), covering nearly all traditionally recognised taxa except *M. melanoleucus*, for which molecular data are unavailable. The ASAP analysis delimited 19–21 molecular operational taxonomic units (MOTUs), depending on the selected model. Overall, the three applied models yielded congruent results, with only one notable exception: the SD and JC69 models grouped two highly supported Costa Rican *M. comisteus* clades and a sister *M. comisteus* clade from Peru into the same MOTU, whereas these clades were differentiated as distinct MOTUs under the K80 model. Notably, molecular-based delimitation methods revealed significantly more entities recognised as potential species than currently accepted by traditional taxonomy species, suggesting the presence of cryptic diversity within the genus. Besides *M. humboldti* and *M. clenchi*, each represented in the phylogenetic analyses by a single specimen, only specimens of *M. impeditus* and *M. votani* were recovered by the ASAP approach as single entities (MOTUs), thus showing assignment consistent with their current taxonomic arrangement. All other species (*M. putridus*, *M. mathani*, *M. pyracmon*, *M. cognatus*, *M. xylotribus*, and *M. comisteus*) were split into 2–4 lineages (MOTUs) by the delimitation analyses.

### 3.2. Taxonomic Overview of the Genus Morpheis

*Morpheis* Hübner, [1820]: 196 [2].

Type species (by subsequent designation): *Phalaena pyracmon* Cramer, [1780]: 169, Pl. 287, Figure 13 [3], designated by Roepke (1957: 18) [4].

*Neocossus* Houlbert, 1916: 89 [6]. [Synonymised by Donahue, 1980: 179 [23].]

Type species (by original designation): *Endoxyla strigillata* Felder, 1874 [7]: Pl. 81, Figure 5.

*Xylotrypa* Turner, 1918: 162 [11]. [Synonymised by Donahue (1980: 179) [23].]

Type species (by monotypy): *Endoxyla strigillata* Felder, 1874: Pl. 81, Figure 5.

**Figure 2:**
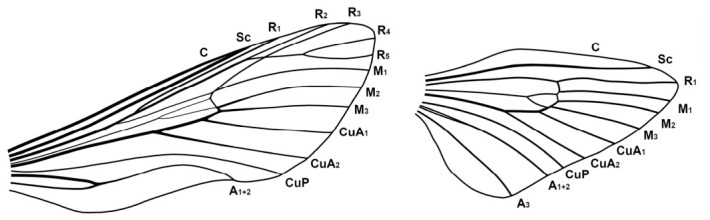
Wing venation of *Morpheis pyracmon* (Cramer, [1780]): forewing (**left**), hindwing (**right**).

**Diagnosis.** Representatives of the genus are characterised by the following traits: imago size varies from medium to very large (wingspan 35–150 mm). The forewing exhibits a longitudinal dark stripe of irregular shape, formed by a thickening of the wavy pattern, starting from the centre of CuA and extending to the wing apex. Most species also possess a similar stripe along basal part of the costal margin, confined below by CuA or M and extending to near the base of R1; in *M. xylotribus* the forewing is characterised by a brownish patternless zone on the costal margin, also confined below by CuA and gradually lightening towards the apex. In general, wing venation follows the typical Zeuzerinae pattern (Figure 2). The genus resembles the New World Zeuzerinae genus *Brypoctia* Schoorl, 1990, sharing common features in external morphology and the genitalia structure: the bipectinate part of the male antenna occupying less than half of its total length; white ground colour of the wings with dark, wavy pattern; gnathos arms broadening into sclerotised, unfused plates; and the presence of a harpe on the ventral side of the valva. At the same time, *Morpheis* is distinguished by the following distinctive diagnostic characters: larger imagoes, with wingspans of 35–150 mm compared to 25–55 mm in *Brypoctia*, a robust thorax and abdomen; a well-developed and consistently present medial cell in the wing; more distinctive and pronounced apices of the fore- and hindwings in males; a more curved dorsal margin of the forewing; more prominent alternating dark spots on the outer margin of the wings at the vein termini.

**Female genitalia.** Papillae anales oval, covered with short setae; ovipositor long, telescopic, with bundles of long hairs in the basal extended part; apophyses posteriores significantly longer than apophyses anteriores; ostium poorly immersed; antrum sclerotised, of irregular shape, ductus short; corpus bursae of asymmetric bag-like shape with distinclty folded surface; inner surface finely spinulose; the inner lateral surface of the lower cenral part of the corpus bursae bears a signum of variable size and diverse, irregular shape, represented by a convex sclerotised area composed of larger spinules; shape of signum varies within the species; thin duct of bulla extends from wall of upper part of bursa; bulla spherical or elliptical, of medium size, with unfolded walls; ductus seminalis thin, extends from lateral lower side of bursa.

**Distribution.** The most widely distributed Zeuzerinae genus in the New World, ranging from the USA (Arizona) and northern Mexico to the Monte Desert in Argentina, excluding the Antilles biogeographic subregion and the Andean highlands.

#### 3.2.1. *Morpheis clenchi* Donahue, 1980

**Figure 3:**
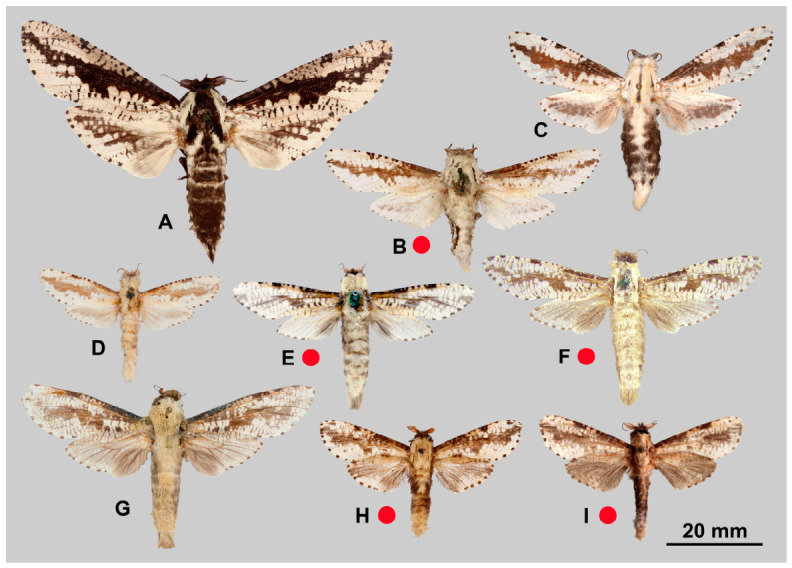
*Morpheis* imagoes: (**A**) *M. clenchi* Donahue, 1980, male, USA, Arizona (NHMUK); (**B**) *M. cognatus* (Walker, 1856), male, holotype, Honduras (NHMUK); (**C**) *M. cognatus* (Walker, 1856), male, Mexico, Chiapas (MWM); (**D**) *M. cognatus* (Walker, 1856), male, Mexico, Misantla (ZSM); (**E**) *M. comisteus* (Schaus, 1911), male, holotype, Costa Rica, Tuis (USNM); (**F**) *M. comisteus* (Schaus, 1911), male, paratype, Costa Rica, Sixola riv. (CMNH); (**G**) *M. comisteus* (Schaus, 1911), male, Venezuela, Bolívar (MWM); (**H**) *M. humboldti* Naydenov, Yakovlev & Penco, 2021, male, holotype, Brazil, Amazonas (NHMUK); (**I**) *M. humboldti* Naydenov, Yakovlev & Penco, 2021, male, paratype, Brazil, Rondônia (MNHN); red circles mark type specimens.

**Figure 4:**
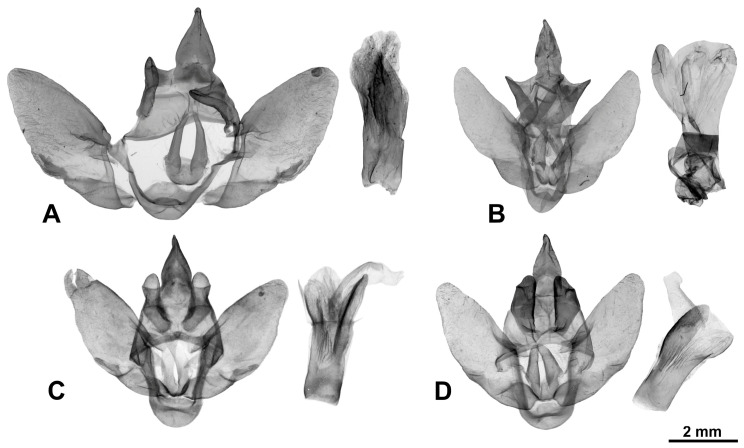
*Morpheis* male genitalia: (**A**) *M. clenchi* Donahue, 1980, USA, Arizona, genitalia slide No. 010315455 (NHMUK); (**B**) *M. cognatus* (Walker, 1856), Mexico, Misantla, genitalia slide No. E10 (ZSM); (**C**) *M. comisteus* (Schaus, 1911), Suriname, Aroewarwa Creek, genitalia slide No. 010315454 (NHMUK); (**D**) *M. humboldti* Naydenov, Yakovlev & Penco, 2021, holotype, Brazil, Amazonas, genitalia slide No. 010315450 (NHMUK).

**Figure 5:**
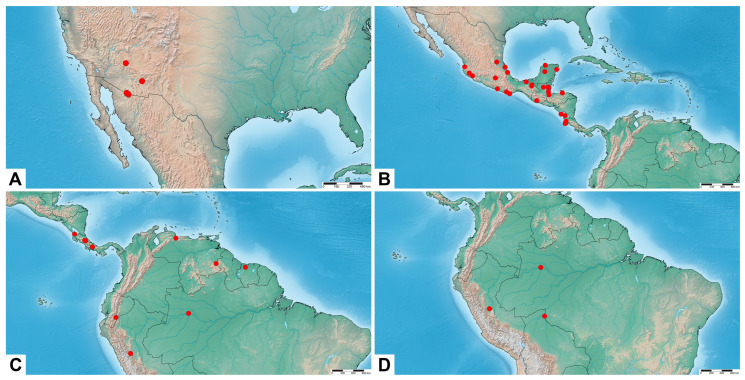
Distributional records of *Morpheis*: (**A**) *M. clenchi* Donahue, 1980; (**B**) *M. cognatus* (Walker, 1856); (**C**) *M. comisteus* (Schaus, 1911); (**D**) *M. humboldti* Naydenov, Yakovlev & Penco, 2021.

Figure 3A, Figure 4A and Figure 5A

**Material examined: USA:** 1♂, Arizona, Oslar, Nogales, slide NHMUK 010315455, NHMUK 012832422 (NHMUK).

**Literature data** (after [23]): Holotype ♂: **USA**, Arizona, Santa Cruz Co., 5 mi W of Peña Blanca, 28 Jul 1973, at ultraviolet black light, leg. R. Wielgus (LACM). Paratypes: **USA**: 2♂, Arizona, Santa Cruz Co., Peña Blanca Lake, Oro Blanco Mountains, 10 air mi WNW of Nogales, alt. 3700 ft, 19 Jul 1976, leg. E.M. Brown & J.S. McElfresh (Brown & McElfresh coll.); 2♂, same locality, 24 Jul 1973, leg. W.A. Harding (LACM); 1♂, same locality, 28 Jul 1973, leg. B. Griffin (Frack coll.); 2♂, same locality, 26 Jul 1973, leg. R.J. Ford (LACM and Arizona State University); 2♂, same locality, 7 Jul 1972, at black light, leg. D.C. Frack (LACM, Frack coll.); 1♂, same locality, 12 Jul 1967, leg. K. Roever (Lloyd M. Martin coll.); 1♂, Arizona, Santa Cruz Co., Campground near Peña Blanca Lake, alt. 3950 ft, 27 Jul 1976, at ultraviolet light, leg. J. Wiseman (LACM); 1♂, Arizona, Sycamore Canyon, 16 Jul 1974, leg. D.G. Marqua (Marqua coll.).

**Diagnosis.** Externally, the species is most similar to *M. cognatus* and *M. mathani*. They are united by a reduced wavy pattern on the fore- and hindwings. From *M. mathani* the species differs in smaller size, a more convex and rounded outer margin of the hindwings, less distinct borders of the longitudinal stripe on the forewings and a well-developed harpe on the valves. From *M. cognatus* it differs in larger size, a black (or dark-brown) wing pattern on a white ground colour, a black central part of the thoracic dorsum contrasting with light tegulae, and a distinctive harpe on the valves in the male genitalia.

**Distribution.** Southwestern United States (Arizona) (Figure 5A).

#### 3.2.2. *Morpheis cognatus* (Walker, 1856)

*Zeuzera cognata* Walker, 1856: 1532 [8]

*Xyleutes (Neocossus) mexicanus* Houlbert, 1916: 88 [6] [Synonymised by Dyar, 1940: 1267 [13].]

Figure 3B–D, Figure 4B and Figure 5B

**Type material examined.** Holotype ♂: **HONDURAS** (NHMUK); type 1♂ of *Xyleutes mexicanus*: **MEXICO**, Veracruz, Misantla, Apr–May 1912, leg. W. Gudelmann (MNHN). **Additional material examined. MEXICO**: 1♂, Misantla, Jul [19]14 (ZSM); 1♂, “Mexica”, 28 Oct [19]16, “Laue” (ZSM); 1♂, Oaxaca, El Sagrado, San Gabriel Mixtepec, alt. 710 m, 18 Jun 2010, leg. P. Schmit (RYB); 1♂, Chiapas, “Palenque Agua Azul”, 16 Mar [19]81, leg. N. Flanger (MWM); 2♂, Colima, “62.24.”, Jun [19]23, “Pres. by Prof. M. Draudt”, “Joicey Coll. Brit. Mus. 1925–157” (NHMUK). **BELIZE**: 1♂, Upper Raspaculo Valley, 16.816667°N, 88.800000°W, alt. 445 m, at light, 8 May 1993, leg. M. Matthews, “Joint Services Scientific Exped. to the Upper Raspaculo 1993” (NHMUK); 1♂, Punta Gorda, Jun 1933, leg. J.J. White (NHMUK); 1♂, Rio Temash, leg. J.J. White (NHMUK). **GUATEMALA**: 1♂, Cayuga, “Mar.”, “1919–266” (NHMUK). **COSTA RICA**: 1♂, Peninsula Nicoya, environs of Sámara, alt. 10–100 m, 11–14 Nov 1995, leg. de Freina (MWM).

**Diagnosis.** Externally, the species is most similar to *M. clenchi* and *M. mathani*. They are united by a reduced wavy pattern on the fore- and hindwings. From *M. mathani* the species differs in smaller size, a continuous longitudinal stripe on the forewings and typically a less contrasting wing pattern. From *M. clenchi* it differs in smaller size, a beige (or light-brown) pattern on a white ground colour, and a poorly developed harpe on the valves in the male genitalia.

**Distribution.** Southern North America: central and southern Mexico. Central America: Belize, Guatemala, Honduras, Costa Rica, and Nicaragua (Figure 5B).

#### 3.2.3. *Morpheis comisteus* (Schaus, 1911)

*Zeuzera comisteon* Schaus, 1911: 628 [16]

Figure 3E–G, Figure 4C and Figure 5C

**Type material examined.** Holotype ♂: **COSTA RICA**, Tuis, Jun, type No. 17302 (USNM). Paratypes: 1♂, **COSTA RICA**, Juan Viñas, Jun (CMNH); 1♂, **COSTA RICA**, Sixola River, Sep (CMNH). **Additional material examined. COSTA RICA:** 1♂, Sixola River, “Mar.” (NHMUK); 1♂, San José, “48. 22.”, “H. Schmidt”, 31 Jan [19]22, “Joicey Coll. Brit. Mus. 1925–157” (NHMUK); 1♂, Tuis, alt. 2500 ft, “Jun”, “W. Schaus”, “1911–32” (NHMUK); 1♂, Orosi, Volcán Irazú, alt. 1200 m, coll. Fassl, Teplitz, “Joicey Coll. Brit. Mus. 1925–157” (NHMUK); 1♂, Sixola River, Sep 1909, “W. Schaus don. 1913, ex Coll. W.S.” (OUMNH). **PANAMA:** 1♂, Lino, alt. 800 m, “Coll. Fassl”, “Joicey Coll.”, “Brit. Mus.”, “1925–157” (NHMUK). **VENEZUELA:** 1♂, Edo. Bolívar, La Escalera, alt. 1420 m, 5.969683° N, 61.420117° W, 24–26 Sep 2011, leg. M. Hiermeier, G. Roiger, W. Stoiber & R. Wanninger, GenPr Heterocera MWM: 36.975, “AN MWM 20” (MWM); 1♂, Benjuma, Casa María, Via Palmichal, alt. 850 m, Nov 1996, leg. N. Flauger (MWM). **SURINAME:** 1♂, Aroewarwa Creek, Marowijne Valley, Jun 1905, leg. S.M. Klages, SLIDE NHMUK 010315454, NHMUK 012832421 (NHMUK). **BRAZIL:** 1♂, Amazonas, Fonte Boa, Aug 1906, leg. S.M. Klages, SLIDE NHMUK 010315451, NHMUK 012832418 (NHMUK). **ECUADOR:** 2♂, Morona Santiago Prov., San Carlos de Limón, 3.192222° S, 78.444444° W, alt. 937 m, 24 Nov 2011, leg. V. Sinyaev & O. Romanov (MWM). **PERU:** 1♂, Huánuco, Santa Rosa, environs of Tingo María, alt. 810 m, 17 Sep 2003, leg. P. Schmit, “ANRYB 95” (RYB).

**Diagnosis.** Externally, the species is similar to *M. humboldti*, *M. melanoleucus*, *M. putridus*, and *M. pyracmon*. They all share a common forewing colouration: a well-expressed wavy transverse pattern on a white ground colour with a continuous longitudinal stripe of irregular shape. From *M. melanoleucus*, *M. putridus* and *M. pyracmon* the species differs in a less contrasting and lighter wavy pattern, the shape of the longitudinal stripe on the forewings (which is especially noticeable in contrast with the darker dots along the outer margin of the wings), and a smoky-grey wing pattern on the hindwings. From *M. putridus* and *M. pyracmon* it differs in the absence of a sharply defined border between the longitudinal stripe and wavy pattern (in several specimens, typically the smaller ones, the outline of the longitudinal stripe may be almost or completely lost toward the apex). From *M. humboldti* the species differs in larger size, more pronounced wing apices (the wings appear more elongated and narrower), a clearer transverse wavy pattern, and a lighter smoky pattern on the hindwings.

**Distribution.** Central America: Costa Rica and Panama. Northern South America: Ecuador, Venezuela, Peru, Suriname, and northern Brazil (Figure 5C).

#### 3.2.4. *Morpheis humboldti* Naydenov, Yakovlev & Penco, 2021 [26]

Figure 3H,I, Figure 4D and Figure 5D

**Type material examined**. Holotype ♂: **BRAZIL**, Amazonas State, Fonte Boa, Jul 1906, leg. S.M. Klages, SLIDE NHMUK 010315450, NHMUK 012832417 (NHMUK). Paratypes: 2♂, **BRAZIL**, same locality (NHMUK); 1♂, **BRAZIL**, Rondônia State, Rio Mamoré, Yata, below Guajará-Mirim, May 1966, coll. Moinier (MNHN); 1♂, **PERU**, Huánuco Province, Yuyapichis, ACP Panguana, alt. 220 m, 9.600000° S, 74.933333° W, Jun 2013, leg. H. Thöny, GenPr Heterocera MWM 36.977 (MWM). **Additional material examined. PERU:** 1♂, Huánuco, Yuyapichis, Fazenda Tropical, 9.616667° S, 74.933333° W, alt. 210 m, Jun 2013, leg. A. Eichinger, “AN MWM 21” (MWM).

**Diagnosis.** Externally, the species is similar to *M. comisteus*, *M. melanoleucus*, *M. putridus*, and *M. pyracmon*. They all share a common forewing colouration: a well-expressed transverse wavy pattern on a white ground colour, with a continuous longitudinal stripe of irregular shape. From all the species mentioned above, *M. humboldti* differs in smaller size and visually more rounded wings without pronounced apices and with a less conspicuous wavy pattern (lacking pronounced longitudinal weaving). From *M. comisteus* the species differs in a more contrasting dark wavy pattern with a distinct longitudinal stripe on the forewings and a darker smoky pattern on the hindwings. The relative reduction in the wavy pattern makes this species similar to *M. cognatus*, although *M. humboldti* is characterised by a less pronounced pattern reduction, and a more diffuse border of the smoky pattern on the hindwings.

**Distribution.** Amazonia: northwestern Brazil and central Peru (Figure 5D).

#### 3.2.5. *Morpheis impeditus* (Wallengren, 1860)

*Phragmataecia impedita* Wallengren, 1860: 44 [14]

*Endoxyla strigillata* C. Felder, 1874: Pl. 81, Figure 5 [7]. [Synonymised by Gaede (1933: 822) [12].]

**Figure 6:**
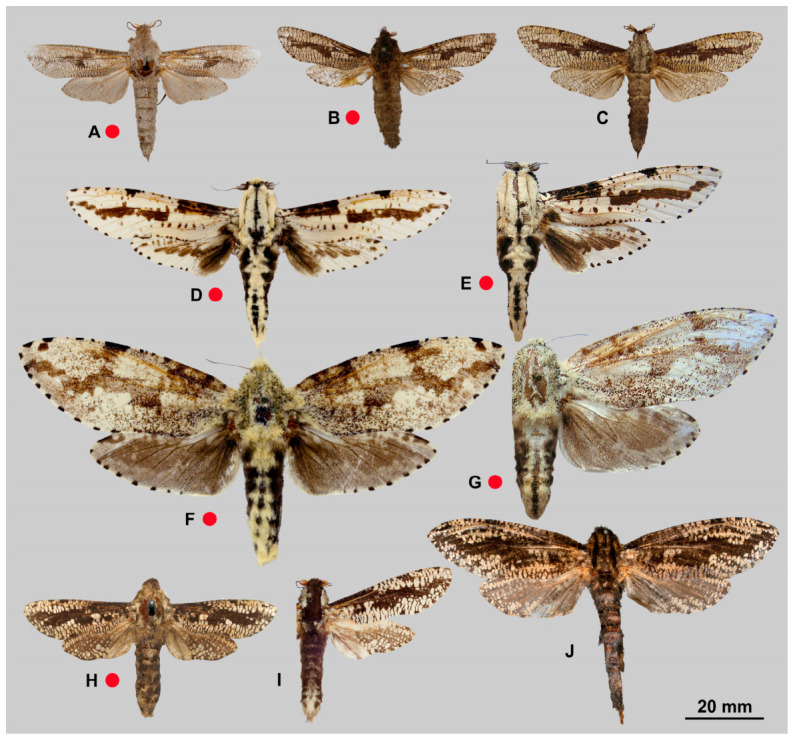
*Morpheis* imagoes: (**A**) *M. impeditus* (Wallengren, 1860), male, holotype (SMNH); (**B**) *M. impeditus* (Wallengren, 1860), male, holotype of *Endoxyla strigillata*, Argentina, La Plata (NHMUK); (**C**) *M. impeditus* (Wallengren, 1860), male Argentina, San Juan (MWM); (**D**) *M. mathani* (Schaus, 1901), male, syntype, Peru, Huambo (USNM); (**E**) *M. mathani* (Schaus,1911), male, syntype of *Xyleutes oberthueri* Houlbert, 1916, Peru, Huambo (MHNH); (**F**) *M. mathani* (Schaus, 1901), female, syntype, Peru, Huambo (USNM); (**G**) *M. mathani* (Schaus,1911), female, syntype of *Xyleutes oberthueri* Houlbert, 1916, Peru, Huambo (MHNH); (**H**) *M. melanoleucus* (Burmeister, 1878), male, holotype, Argentina, Buenos Aires (MACN); (**I**) *M. melanoleucus* (Burmeister, 1878), male, Argentina, Cordoba (ZSM); (**J**) *M. melanoleucus* (Burmeister, 1878), female, Argentina, Cordoba (ZSM); red circles mark type specimens.

**Figure 7:**
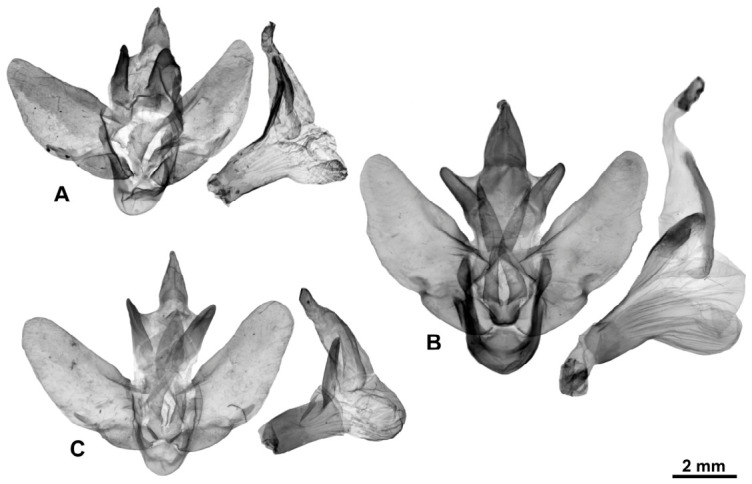
*Morpheis* male genitalia: (**A**) *M. impeditus* (Wallengren, 1860), Argentina, San Juan, genitalia slide No. 26.719 (MWM); (**B**) *M. mathani* (Schaus, 1911), Peru, Madre de Dios, genitalia slide No. 28.487 (MWM); (**C**) *M. melanoleucus* (Burmeister, 1878), Argentina, Cordoba, genitalia slide No. 26.718 (MWM).

**Figure 8:**
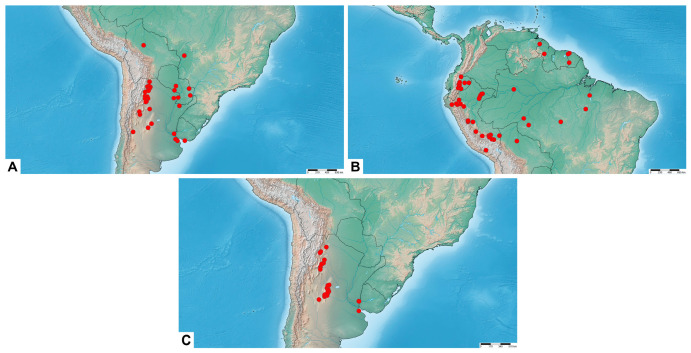
Distributional records of *Morpheis*: (**A**) *M. impeditus* (Wallengren, 1860); (**B**) *M. mathani* (Schaus, 1911); (**C**) *M. melanoleucus* (Burmeister, 1878).

Figure 6A–C, Figure 7A and Figure 8A

**Type material examined.** Holotype ♂: [without locality data] (SMNH). Holotype ♂ of *Endoxyla strigillata*, **ARGENTINA**, La Plata, NHMUK 012832608 (NHMUK). **Additional material examined. ARGENTINA:** 14♂, Jujuy, Santa Barbara Mts., 12 km SW Palma Sola, Eco Portal de Piedra NP, 24.095167° S, 64.399139° W, alt. 1045 m, 29 Oct–2 Nov 2019, leg. R.V. Yakovlev (RYB); 4♂, Jujuy, 25 km NE Palma Sola, 23.814917° S, 64.212417° W, alt. 454 m, 23 Oct 2019, leg. R.V. Yakovlev (RYB); 7♂, Jujuy, 10 km SEE Caimancito, 23.744056° S, 64.519222° W, alt. 405 m, 25–26 Oct 2019, leg. R.V. Yakovlev (RYB); 2♂, Jujuy Prov., Santa Barbara Mts., 12 km SW Palma Sola, Eco Portal de Piedra NP, 24.095139° S, 64.399111° W, alt. 1045 m, 19–22 Oct 2019, leg. R.V. Yakovlev (RYB); 4♂, Jujuy, Route Santa Clara–Palma Sola km 17, alt. 1180 m, 20 Nov 2003, leg. P. Schmit (RYB); 1♂, Jujuy, Rte PN, Calilegua–Valle Grande km 7, alt. 1580 m, 23 Nov 2003, leg. P. Schmit (RYB); 2♂, Jujuy, Dto. Ledesma, Yuto (ASP) El Pantanoso, 27–30 Oct 2021, leg. L. Aguado (LA); 1♂, Jujuy, Villamonte, Eco-Portal de Piedra, Jan 2013, leg. L. Aguado, “Prep Gen FCP No. 8” (FCP); 1♂, Jujuy, Las Lancitas, 13 Jan 2016, leg. P. Loescher (FCP); 1♂, Jujuy, “Yutu”, 349 m, 11 Feb [19]55, leg. Juan Foerster (ZSM); 1♂, La Rioja, leg. Giacomelli, “coll. Cte. Hartig” (ZSM); 1♂, La Rioja, Anillaco, 30 Nov 1998, coll. O. Di Iorio (FCP); 1♂, La Rioja, 3 Dec 1998 (FCP); 2♂, La Rioja, 10 Dec 1998 (FCP); 1♂, La Rioja, 20 Dec 1998 (FCP); 1♂, La Rioja, 1 Jun 1999 (FCP); 2♂, La Rioja, 4 Jun 1999 (FCP); 1♂, Salta, Route La Vica–Pampa Grande km 26, alt. 1600 m, 15 Nov 2003, leg. P. Schmit (RYB); 5♂, Salta, coll. A. Breyer (MLP); 1♂, Salta, Orán, Aguas Blancas, Jan 1999, leg. E. Nuñéz Bustos (ENB); 1♂, Salta, Dec 1943, leg. F. Monros (FML); 1♂, Salta, Rosario de la Frontera, Feb 1935 (FML); 1♂, Salta, Feb 1936 (FML); 1♂, Salta, El Morenillo, Feb 1936, “41” (FML); 1♂, Santiago del Estero, Depto. Choya, Laprida, 12 Dec 2009, leg. A. Borquez (RYB); 1♂, San Juan, [Fenili], “J.R”, 9 Jan [19]94, “GenPr-Heterocera MWM 26.719” (MWM); 1♂, Mendoza, Dto. Guaymallén, Los Corralitos, alt. 650 m, 30 Dec 2010, leg. G. Gomariz (CUYO); 1♂, Tucumán, leg. Steinbach, SLIDE NHMUK 010315449, NHMUK 012832416 (NHMUK); 1♂, Tucumán, leg. Schreiter (FML); 1♂, Tucumán, 27 Feb 1918, “49”, “*Xyleutes albogrisea* Dognin (det. P. Gentili)” (FML); 1♂, Tucumán, 20 Feb 1923 (FML); 1♂, Tucumán, San Pedro de Colalao, Depto. Trancas, Dec 1952, leg. Arnau (MACN); 1♂, Tucumán, Quebrada de Lules, Jan 1922 (FML); 1♂, Tucumán, Feb 1922 (FML); 1♂, Tucumán, Feb 1925 (FML); 1♂, Tucumán, Mar 1925 (FML); 1♂, Tucumán, Jan 1926 (FML); 1♂, Tucumán, Feb 1926 (FML); 1♂, Córdoba, San Javier, La Paz, 15–31 Dec 1928, coll. C. Bruch (MLP); 1♂, Córdoba, coll. P. Köhler (MLP); 1♂, Misiones, “MACN-Bar-Lep-ct 0762” (MACN); 1♂, Entre Ríos, Ceibas, 10 Dec 1991, leg. O. Di Iorio (FCP); 1♂, Entre Ríos, Aristóbulo del Valle, Camping del Valle del Cuñá Pirú, Nov 2014, leg. L. Aguado (FCP); 1♂, Buenos Aires, Capital Federal, Dec 1942 (MACN); 1♀, Buenos Aires, Vicente Lopez, 2 Jan 1953, coll. Petrovsky (MLP); 1♂, Buenos Aires, 18 Dec 1951, coll. Petrovsky (MLP); 1♂, Buenos Aires, Olivos, 31 Dec 1950, coll. A. Breyer (MLP); 1♂, Buenos Aires, “Dem”, coll. Bolle, leg. L. Aguado (FCP); 2♂, Buenos Aires, 29 Dec 1955 (MACN); 1♀, Buenos Aires, “5569” (MACN); 1♀, Buenos Aires (MACN); 1♀, Buenos Aires, “11948” (MACN); 1♀, Buenos Aires, 1st Section, Paraná River Delta, 27 Jan 2006, leg. R. Schincariol & G. Mujica (IMZA); 1♂, Buenos Aires, E.E. Delta, 4 Feb 2006, leg. Ing. Alva (IMZA); 1♀, Buenos Aires, San Miguel, 26 Nov 1940, leg. Bridarolli, ex coll. O. Di Iorio (FCP). **URUGUAY:** 1♀, Montevideo, Nov 1943, “*Morpheis xylotribus*, F. Schweizer det.” (MHNU). **BOLIVIA:** 2♂, Chocolatal, May 2017, leg. A. Borquez, ex coll. F.C. Penco, “DNA preparation №6”, “ANRYB 98” (RYB). **PARAGUAY:** 1♂, Pte. Hayes Dep., Costa Esmeralda, 23.666667° S, 58.483333° W, 4–8 Nov 2011, leg. U. Drechsel (RYB); 1♂, same locality, 10–14 Nov 2013, leg. U. Drechsel (RYB); 1♂, Alto Parana Dep., Estancia Dimas, 25.550000° S, 55.216667° W, 16 Nov 2011, leg. U. Drechsel, “ANRYB 101” (RYB); 1♂, same locality, 24 Mar 2011, leg. U. Drechsel (RYB); 1♂, Canindeyu Dep., Mbaracayu, 24.133333° S, 55.516667° W, 13–15 Nov 2017, leg. U. Drechsel (RYB); 1♂, Ñeembucú Dep., Zanjita, 26.050000° S, 57.933333° W, 18–19 Feb 2017, leg. U. Drechsel, “DNA preparation №8” (RYB).

**Diagnosis.** Externally, the species is similar to *M. putridus* and *M. pyracmon*. They are united by a light-brown (or beige) colour of the rami in males and a common wing pattern type: a uniformly expressed streaky transverse pattern on a light ground colour, with a continuous longitudinal stripe of irregular shape. From all the species listed above, *M. impeditus* differs in grey ground colour of the fore- and hindwings, which makes specimens appear darker overall, and in the more uniform width of the wavy pattern strokes.

**Distribution.** Bolivia, southwestern Brazil, Paraguay, Uruguay, and northern Argentina (Figure 8A).

**Remarks.** M. Gaede [12] noted that the type locality of *M. impeditus* was erroneously given as Australia and stated that *M. impeditus* and *M. strigillatus* belong to the same species. J.P. Donahue [23], in his review of the genus, treated these two taxa as distinct species. After examining the type specimens of both taxa, we follow M. Gaede’s opinion and confirm the synonymy of *M. strigillatus* and *M. impeditus*.

#### 3.2.6. *Morpheis mathani* (Schaus, 1901)

*Duomitus mathani* Schaus, 1901: 45 [17]

*Xyleutes (Neocossus) oberthueri* Houlbert, 1916: 86–88 [6] [Synonymised with *Xyleutes cognata* by Dyar, 1940: 1267 [13]; revised synonymy by Donahue (1980: 179) [23].]

*Xyleutes cognatus distinctus* Bryk, 1953: 267 [25] [Synonymised by Donahue (1980: 179) [23].]

Figure 6D–G, Figure 7B and Figure 8B

**Type material examined.** Syntypes: 1♂, **PERU**, Huambo, M. de Mathan, “IVe Trim. 1889”, “Collection WmSchaus”, “type No. 12568 USNM”, “USNMENT 01198236” (USNM); 1♀, same data, “USNMENT 01198237” (USNM). Syntypes of *Xyleutes oberthueri*: 1♂, same data, “Coll. R. Biedermann”, “ex-coll. Ch. Oberthur” (MHNH); 1♀, same data, “allotype” (MHNH). **Additional material examined. PERU**: 1♂, Huánuco Province, Tingo María, 5 Jan 2002, leg. D. Pecherskyi (RYB); 4♂, Huánuco Province, Her. Valizan, 30 km Tingo María, alt. 1170 m, 18 Sep 2003, leg. P. Schmit, “P03-03-18” (RYB); 1♂, Huánuco Province, 26 km SW of Tingo María, Pte Cayumba, alt. 750 m, 11 Dec 2014, leg. A. Sokolov (RYB); 3♂, Huánuco Province, Santa Rosa, environs of Tingo María, alt. 810 m, 17 Sep 2003, leg. P. Schmit, “P03-02-17” (RYB); 1♂, Huánuco Province, Satipo, Tingo María, 1999, leg. H. Thöny (MWM); 7♂, Huánuco Province, Yuyapichis, ACP Panguana, alt. 220 m, 9.600000° S, 74.933333° W, Jun 2013, leg. H. Thöny, “AN MWM 7” (MWM); 1♂, same locality, May 2013, alt. 220 m (MWM); 1♂, Huánuco Province, Yuyapichis, Faz. Tropical, 9.616667° S, 74.933333° W, alt. 210 m, Mar 2013, leg. A. Eichinger, “AN MWM 8” (MWM); 1♂, Junín Region, Satipo Province, San Martín de Pangoa District, 11.704700° S, 74.301600° W, alt. 960 m, 22 Jan 2017, leg. A. Kozlov & Yu. Kovaleva (RYB); 2♂, Amazonas Dept., Utcubamba Prov., Montenegro, alt. 900 m, Dec 2007, coll. R. Brechlin (MWM); 2♂, Amazonas Dept., Puente Nieva, alt. 950 m, Jun 2007, leg. R. Marx (MWM); 2♂, San Martín Dept., El Carrizal, alt. 2000 m, 1–12 Oct 2010, ex coll. R. Brechlin (MWM); 1♂, Amazonas, Utcubamba Prov., Puente Nieva, 4.833333° S, 77.950000° W, alt. 800 m, Dec 2007 (MWM); 7♂, Amazonas, Utcubamba Prov., Montenegro, alt. 900 m, Dec 2007 (MWM); 2♂, San Martín Dept., Mina de Sal, alt. 1400 m, 2007, leg. R. Marx (MWM); 2♂, Cusco Prov., Río Unebamba, Koribeni, alt. 700 m, 1–5 Dec 1994, leg. Hácz & Juhánz (MWM); 5♂, Camicana–Chico, Río Carbon, Madre de Dios, Manu Park, alt. 800–1000 m, 3 Apr 1998 (MWM); 1♂, Iquitos, Marañón R., Nanta District, Buen Fin, 6–7 Feb [19]97 (MWM); 1♂, S of Iquitos, Tavayo R., 31 Jan [19]97 (MWM); 23♂, Madre de Dios Dept., Route Salvación–Pillahuata, Manu Park, alt. 800–2500 m, Oct–Nov 1998, via R. Marx, ex coll. A. Shintlmeister (MWM); 7♂, Cusco Dept., Chontachaca, Manu Park, alt. 800 m, Jan 1999, via R. Marx, ex coll. A. Shintlmeister (MWM); 3♂, Piura Dept., Abra Porcuya, alt. 1800 m, May 2007 (MWM); 1♂, Montenegro, Río Marañón, 7 Oct 1961, leg. Williner (FCP); 3♂, Río Huallaga, alt. 670 m, Oct 1947, leg. Weyrauch (FML); 1♂, San Gabán, Parao, alt. 2300 ft, Mar–Apr 1913, “1914-180.” (NHMUK); 1♂, N Peru, Rentema Falls, Upper Marañón, alt. 1000 ft, A. & E. Pratt (NHMUK); 1♂, “1 Sep–5 Nov 1923, ix–v incl., Dry S. Ecuador–Peru Oriente Andes foothills and just east, 5–800 ft, R.H. Thomas coll., d.d. 1923”, “Capt. along lower Morona and Santiago Rivers, Marañon River (Upper Amazon) between their mouths on N (left) bank”, “1923”, “1158” (OUMNH). **ECUADOR:** 1♂, Morona Santiago Prov., Río Bamba–Macas, 2.213889° S, 78.138889° W, alt. 1200 m, 29 Mar 2012, leg. V. Sinyaev & R. Brechlin (MWM); 1♀, “Ecuador” (NHMUK). **GUYANA:** 1♂, Potaro, Jan 1908, leg. S.M. Klages (NHMUK); 1♂, “August”, “71 Baramita”, N.W. District, Guyana, leg. B.F. Coles, “Exp. to Guyana”, “Brit. Mus. 1974-551” (NHMUK). **BRAZIL:** 2♂, Rondônia, vicinity of Cacaulândia, Rancho Grande, alt. 350 m, 1–26 Mar 1999, leg. R. Alves dos Santos (MWM); 1♂, Amazonas, Fonte Boa, Aug 1906, leg. S.M. Klages (NHMUK). **BOLIVIA:** 1♂, Beni Dept., Santa Ana de Yacuma, 13.744444° S, 65.426944° W, Jan 2014, leg. A. Borquez (FCP).

**Diagnosis.** Externally, the species is most similar to *M. clenchi* and *M. cognatus*. They are united by a reduced wavy pattern on the fore- and hindwings. From *M. clenchi* and *M. cognatus*, *M. mathani* differs in the following traits: larger size; an interruption (present in most specimens) between the proximal costal part and the central distal part of the forewing longitudinal stripe; and a more pronounced overall contour of the stripe and the wavy pattern strokes. In male genitalia the species differs from *M. clenchi* in reduced harpe on the valves.

**Distribution.** Northern South America: Guyana, French Guiana, northern Brazil, Ecuador, Peru, and northern Bolivia (Figure 8B).

#### 3.2.7. *Morpheis melanoleucus* (Burmeister, 1878)

*Endoxyla melanoleuca* Burmeister, 1878: 407 [9]

**Figure 9:**
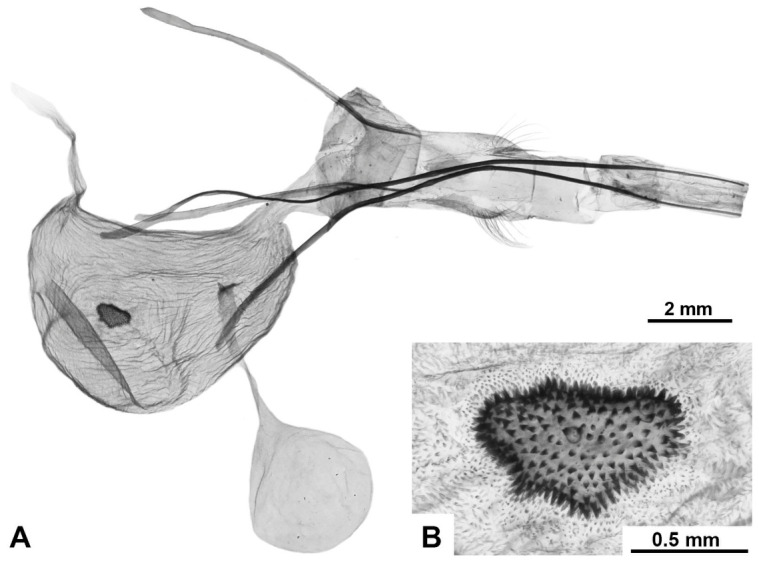
Female genitalia of *Morpheis melanoleucus* (Burmeister, 1878), Argentina, Cordoba, genitalia slide No. 1445 (ZSM): (**A**) general view; (**B**) signum, enlarged.

Figure 6H–J, Figure 7C, Figure 8C and Figure 9

**Type material examined.** Holotype ♂: **ARGENTINA**, Buenos Aires, coll. Antigua (MACN). **Additional material examined. ARGENTINA:** 1♂, Jujuy, Villamonte, Portal de Piedra, Jan 2013, leg. L. Aguado, “Prep Gen FCP No. 7” (FCP); 1♂, Córdoba, Tulumba, 19 Jan [19]94, “J.R.”, “GenPr-Heterocera MWM 26.718” (MWM); 1♂, Córdoba, Salsipuedes, 1 Feb 1937, [leg.] Prof. Dr. C. Hosseus (ZSM); 1♀, Córdoba, Alta Gracia, coll. Hubrich (ZSM); 1♂, Córdoba, Cabaña, 2 Jan 1926 (MLP); 4♂, Córdoba, leg. P. Köhler (MLP); 1♂, Córdoba, Villa Gral. Belgrano, 1–8 Dec 2003, leg. D. Penner (EGO); 8♂, Córdoba, La Paz, 1–15 Jan 1929, coll. C. Bruch, “Ref: 44491” (MACN); 1♂, Córdoba, San Javier, 15–31 Dec 1928, coll. C. Bruch (MLP); 1♂, Córdoba, Anisacate, Dec 1958, leg. Hernandez (MACN); 1♂, Córdoba, El Manzano, Jan 1942, leg. Rodríguez (MACN); 2♂, Córdoba, Alta Gracia, La Granja, “Sierras de Córdoba”, coll. C. Bruch (MLP); 1♀, Córdoba (IMZA); 1♀, Córdoba, Capilla del Monte, Jan 1966, as “*Xyleutes strigillata*”, Rizzo leg., ex coll. H.F. Rizzo (LA); 1♂, Córdoba, 3 Jan 2009, leg. L. Aguado, “Prep Gen FCP No. 11” (FCP); 4♂, Córdoba, Calamuchita, El Sauce, leg. Viana (MACN); 1♂, Córdoba, Dean Funes, 9 Dec 2009, leg. L. Aguado, “Prep Gen FCP №3” (FCP); 1♂, Tucumán, Mar 1905, leg. Steinbach, SLIDE NHMUK 010315453, NHMUK 012832420 (NHMUK); 1♂, Tucumán, Dec 1948 (FML); 1♂, Tucumán, Macomila, Jan 1950, leg. M.A. Teran (FML); 3♂, Tucumán, Concepción, Dec 1946, “*Dumitus pyracmonides* Gentili det.”, leg. Golbach (FML); 1♂, Tucumán, Dique Los Pizarros, 10–13 Dec 1982, “*Dumitus pyracmonides* Gentili det.”, leg. R. Golbach (FML); 1♂, Tucumán, Quebrada de Lules, “30”, 29 Jan 1922 (FML); 1♂, Entre Ríos, Gualeguaychú, Salto de Méndez, 14 Nov 1998 (ENB); 1♂, Salta, coll. A. Breyer (MLP); 1♂, Salta (IMZA); 1♂, Salta, Depto. Rosario de la Frontera, El Morenillo, Mar 1936 (FML); 2♂, Salta, Rosario de Lerma, Apr 1988, coll. Carpintero (CFA).

**Diagnosis.** Externally, the species is most similar to *M. putridus* and *M. pyracmon*. They are united by a light-brown or beige colour of the rami in males, as well as by a common wing colouration pattern: a distinct transverse wavy pattern on a light ground colour with a continuous longitudinal stripe of irregular shape. From *M. putridus* and *M. pyracmon* the species differs in a contrasting wing pattern: a diffused transverse dark-brown stripe and a wavy dark-brown pattern of strokes of varying thickness (generally thick), which in some specimens become significantly transversely intertwined. In the vast majority of specimens, vertex and central part of thoracic dorsum characteristically black, sharply contrasting with light tegulae. In female genitalia, the species is distinguished by a larger bulla and signum relative to the bursa.

**Distribution.** Northern Argentina (Figure 8C).

#### 3.2.8. *Morpheis putridus* (Percheron, 1838) stat. rev.

*Zeuzera putrida* Percheron, 1838: Pl. 4, Figure 1 [18]

*Xyleutes discreta* Dyar, 1940: 1267 [13] **syn. nov.**

**Figure 10:**
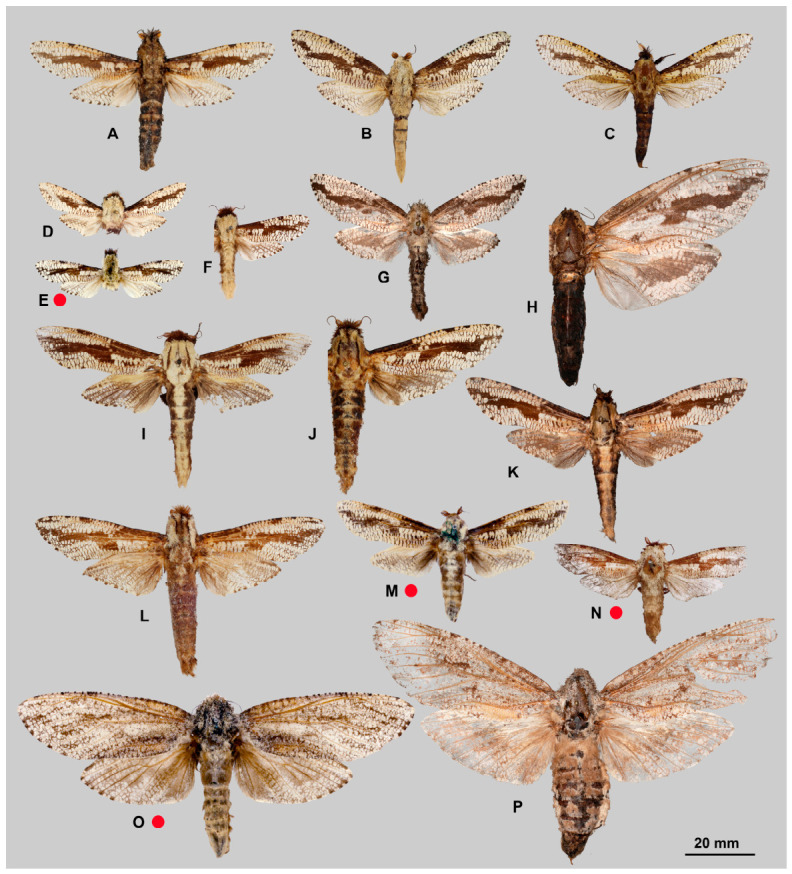
*Morpheis* imagoes: (**A**) *M. putridus* (Percheron, 1838), male, Brazil, Bahia (MWM); (**B**) *M. putridus* (Percheron, 1838), male, Paraguay, Amambay (RYB); (**C**) *M. putridus* (Percheron, 1838), male, Paraguay, Alto Parana (RYB); (**D**) *M. putridus* (Percheron, 1838), male, Brazil, Bahia (MWM); (**E**) *M. putridus* (Percheron, 1838), male, holotype of *Xyleutes discreta* Dyar, 1940, “Bresil” (USNM); (**F**) *M. putridus* (Percheron, 1838), male, Brazil, Santa Catarina (NHMUK); (**G**) *M. putridus* (Percheron, 1838), female, Brazil, Minas Gerais (MWM); (**H**) *M. putridus* (Percheron, 1838), female, Brazil, Espírito Santo (MWM); (**I**) *M. pyracmon* (Cramer, [1780]), male, Suriname (NHMUK); (**J**) *M. pyracmon* (Cramer, [1780]), male, Venezuela, Yaracuy (MWM); (**K**) *M. pyracmon* (Cramer, [1780]), male, Peru, Amazonas (MWM); (**L**) *M. pyracmon* (Cramer, [1780]), male, Mexico, Puebla (MWM); (**M**) *M. pyracmon* (Cramer, [1780]), male, holotype of *Duomitus pyracmonides* Schaus, 1901, Mexico, Orizaba (USNM); (**N**) *M. pyracmon* (Cramer, [1780]), male, holotype of *Zeuzera fracta* Walker, 1856 (NHMUK); (**O**) *M. pyracmon* (Cramer, [1780]), female, holotype of *Zeuzera lelex* Dognin, 1891, Venezuela, Merida (USNM); (**P**) *M. pyracmon* (Cramer, [1780]), female, Colombia, Magdalena (MWM); red circles mark type specimens.

**Figure 11:**
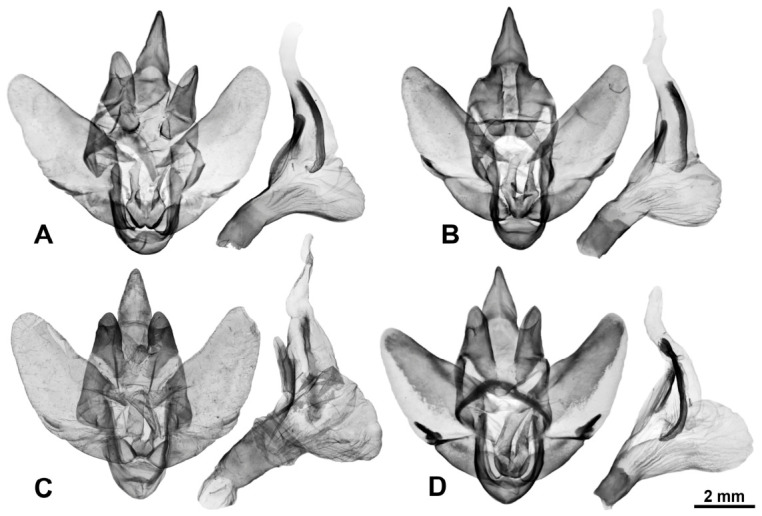
*Morpheis* male genitalia: (**A**) *M. putridus* (Percheron, 1838), Brazil, Espírito Santo, GenPr Heterocera MWM 28.484 (MWM); (**B**) *M. putridus* (Percheron, 1838), Brazil, Minas Gerais, GenPr Heterocera MWM 28.485 (MWM); (**C**) *M. pyracmon* (Cramer, [1780]), Suriname, SLIDE NHMUK 014333367 (NHMUK); (**D**) *M. pyracmon* (Cramer, [1780]), Venezuela, Carabobo, GenPr Heterocera MWM 28.482 (MWM).

**Figure 12:**
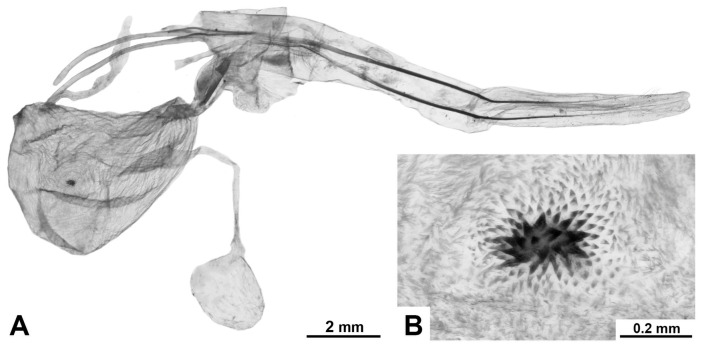
Female genitalia of *Morpheis putridus* (Percheron, 1838), Brazil, Minas Gerais, genitalia slide No. 1341 (MWM): (**A**) general view; (**B**) signum, enlarged.

**Figure 13:**
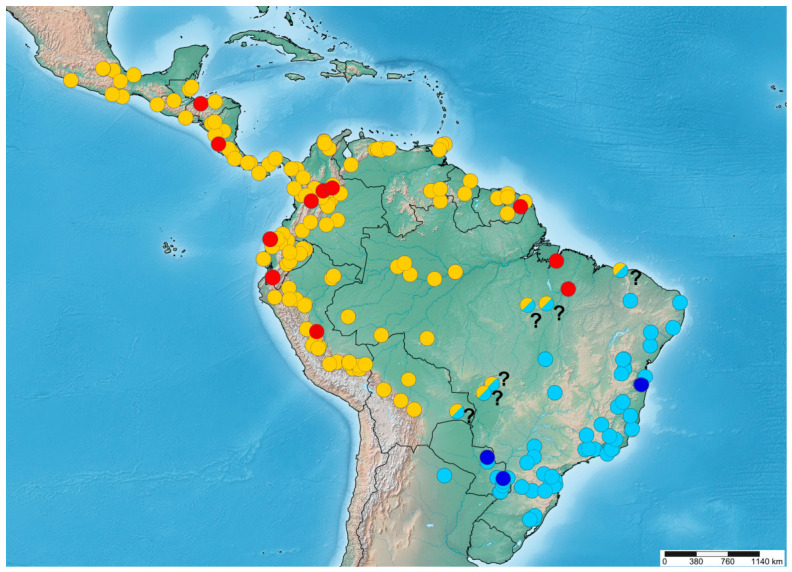
Distributional records of *Morpheis putridus* (Percheron, 1838) and *M. pyracmon* (Cramer, [1780]). Blue circles indicate specimens identified as *M. putridus* by molecular data; light blue circles indicate specimens tentatively identified as *M. putridus*; red circles indicate specimens identified as *M. pyracmon* by molecular data; orange circles indicate specimens tentatively identified as *M. pyracmon*; circles marked with a question mark denote localities situated at the boundary between two biogeographic subregions where species assignment remains uncertain.

Figure 10A–H, Figure 11A,B, Figure 12 and Figure 13

**Material examined (identified by molecular data). BRAZIL:** 2♂, Bahia, Camacan–Serra Bonita, 15.383333° S, 39.550000° W, alt. 800 m, 23 Sep–14 Oct 2011, leg. Schintlmeister & Becker, ex coll. K. Martini, “AN MWM 9” (MWM); 2♂, Bahia, env. Camacan, Fazenda Paris, 15.400000° S, 39.483333° W, alt. 250 m, Jul–Sep 2011, leg. H. Thöny & Greifenstein, “GenPr-Heterocera MWM 28.656”, “AN MWM 10” (MWM). **PARAGUAY:** 1♂, Amambay Dep., Estancia Lai Rahue, 23.333333° S, 56.300000° W, 23–27 Nov 2013, leg. U. Drechsel, “DNA preparation №7” (RYB); 1♂, Alto Paraná Dep., Estancia Dimas, 25.550000° S, 55.216667° W, 16 Nov 2011, leg. U. Drechsel, “ANRYB 78” (RYB).

**Tentatively identified as *M. putridus*: type material examined.** Holotype ♂ of *Xyleutes discreta*: “Bresil”, “Dognin collection”, “type No. 41615 USNM”, “USNMENT 01198203”, “Genitalia Slide by PG-2097 USNM 85537” (USNM). **Additional material examined. BRAZIL:** 1♂, Bahia, Umgebung Camacan, alt. 750 m, 10–14 Nov 2010, leg. Greifenstein Th. (MWM); 5♂, Bahia, Barra da Estiva, Jussiape, alt. 650 m, 10–20 Apr 1997, leg. H. Thöny (MWM); 3♂, Bahia, Chapada, Lençóis, alt. 400 m, Jan–Feb 1998, leg. H. Thöny (MWM); 1♂, Bahia, “Juszlape nördl. von Brumado”, alt. 700 m, Oct 1997, leg. H. Thöny (MWM); 1♂, Bahia, Cipo, alt. 700 m, Nov 1999, leg. H. Thöny (MWM); 1♀, Boca do Mato, Cachoeiras de Macau, 20–30 Jan 1997 (MWM); 2♂, 1♀, Rio de Janeiro, Itatiaia, alt. 800–1200 m, Feb [19]60, leg. H. Ebert (ZSM); 1♂, 1♀, Minas Gerais, Pote, alt. 500 m, 15 Sep–15 Oct 1996, leg. H. Thöny (MWM); 1♂, same locality, 20 Feb 1996, leg. H. Thöny (MWM); 6♂, same locality, 15 Mar 1996, leg. H. Thöny (MWM); 1♂, same locality, 30 Oct 1996, leg. H. Thöny (MWM); 6♂, same locality, 15 Sep–15 Oct 1996, leg. H. Thöny (MWM); 6♂, same locality, 20 Dec 1996–25 Jan 1997, leg. H. Thöny (MWM); 2♂, same locality, 12 Nov 1998, leg. H. Thöny (MWM); 2♂, Minas Gerais, Pote, alt. 300 m, 18–20 Mar 1994, leg. H. Thöny (MWM); 2♂, Minas Gerais, Malacacheta, alt. 500 m, Jan 1998, leg. H. Thöny (MWM); 1♂, same locality, Feb 1997, leg. H. Thöny (MWM); 1♂, Minas Gerais, alt. 300 m, 12–30 Nov 1995, leg. Greifenstein (MWM); 1♂, Minas Gerais, Conceição d’ Ouros, Rte Braseipolis, alt. 920 m, “B04-01-14”, 14 Jan 2004, leg. Pierre Schmit (RYB); 1♂, São Paulo, Martinópolis, Fz Sto Antônio, alt. 420 m, “B04-14-27”, 27 Jan 2004, leg. Pierre Schmit (RYB); 29♂, Espírito Santo, Santa Leopoldina, Dorf Tirol, alt. 700 m, 22–31 Oct 1996, leg. H. Thöny (MWM); 1♂, same locality, 10–25 Nov 1996, leg. H. Thöny (MWM); 5♂, 1♀, same locality, 8–20 Dec 1996, leg. H. Thöny (MWM); 2♂, same locality, 15 Dec 1996, leg. H. Thöny (MWM); 2♂, 1♀, same locality, 20 Feb–20 Mar 1997, leg. H. Thöny (MWM); 9♂, same locality, 20 Mar–Apr 1997, leg. H. Thöny (MWM); 33♂, same locality, 15 May 1997, leg. H. Thöny (MWM); 1♂, same locality, 15 Sep 1997, leg. H. Thöny (MWM); 7♂, same locality, Jan 1998, leg. H. Thöny (MWM); 1♂, same locality, Nov 1998, leg. H. Thöny (MWM); 2♂, same locality, Jan 1999, leg. H. Thöny (MWM); 1♂, same locality, Mar 1999, leg. H. Thöny (MWM); 2♂, Espírito Santo, Santa Leopoldina, Biriricas, alt. 700 m, Jan–Feb 1998, leg. H. Thöny (MWM); 1♂, same locality, 15 Nov 1997, leg. H. Thöny (MWM); 8♂, Espírito Santo, Santa Leopoldina, Boquerão, alt. 600 m, 15 Sep 1997, leg. H. Thöny (MWM); 3♂, same locality, Aug 1997, leg. H. Thöny (MWM); 1♂, Espírito Santo, C. Tirol, alt. 600 m, Feb 2001, leg. K. Martini (MWM); 1♂, Espírito Santo, Tirol, alt. 600 m, Sep 2002, leg. K. Martini (MWM); 1♂, Espírito Santo, May 1921 (FML); 1♂, Rio Grande do Sul, Porto Alegre, “Franz Daniel München” (ZSM); 1♂, Rio Grande do Sul, Hamburgo Velho, Ertl C. (ZSM); 1♂, [Santa Catarina], Treze Tílias, alt. 700–800 m, 8–10 Dec 1997, leg. H. Thöny (MWM); 1♂, [Santa Catarina], Joinville (ZSM); 1♂, [Santa Catarina], Joinville, Feb 1923 (FML); 1♀, Santa Catarina, Treze Tílias, 26.966667° S, 51.416667° W, alt. 980 m, Mar 1999, leg. H. Thöny (MWM); 1♂, Santa Catarina, São Bento do Sul, Serra Rio Natal, alt. 850 m, Nov 1998, leg. H. Thöny (MWM); 1♂, same locality, Apr 1999, leg. H. Thöny (MWM); 1♂, Santa Catarina, Neu Bremen, Rio Laeiss, Jan 1932, coll. F. Hoffmann, “NHMUK 012832419”, “SLIDE NHMUK 010315452” (NHMUK); 2♂, Paraná, Curitiba, Serra do Mar, Estrada de Castelhanos, 30 Nov 1997, alt. 500 m, leg. H. Thöny (MWM); 1♂, Paraná, Ponta Grossa, 28 Dec 1957, “34” (FML); 1♂, “Pará”, [leg.] A.M. Moss (NHMUK); 1♂, Alcobaça, “R. Tocantins”, April, [leg.] H. Fassl (NHMUK); 1♂, Mato Grosso, alt. 2253 ft, “39. 27.”, “Burity, 30 miles N.E. of Cuyabá”, 22–30 Sep [19]27, “C. L. Collanette”, “At light” (NHMUK); 1♂, Petrópolis, alt. 3000 ft, 23 Dec [19]57, “40031”, leg. E.P. Wiltshire (NHMUK); 1♂, Rio de Janeiro, Teresópolis, alt. 3000 ft, 14–18 Dec [19]57, [leg.] E.P. Wiltshire, “Wiltshire Coll. B.M. 1979–433” (NHMUK); 1♂, “Guapí-Mirim, 160 m alt. (Caneca Fina—Rio Sucavão)”, “Mun. Magé—Estado do Rio”, “15 Oct 1977”, “Col. Pearson” (NHMUK); 2♂, “Corcovado”, “700 H”, “Feb 1910”, “Ex Coll. Herbert Druce, 1913”, “Joicey Coll.”, “Brit. Mus. 1925–157” (NHMUK); 1♂, Sta. Catharina, Jaraguá do Sul, Dec 1934, [leg.] F.H. Hoffmann (NHMUK); 1♂, Paraná, Iguaçu, 30 Oct [19]21, “Doncaster private coll., purch. 1927” (OUMNH). **PARAGUAY:** 8♂, Alto Paraná Dep., Estancia Dimas, 25.550000° S, 55.216667° W, 16 Nov 2011, leg. U. Drechsel (RYB); 1♂, same locality, 24 Mar 2011 (RYB); 1♂, same locality, 30 Sep–2 Oct 2012 (RYB). **ARGENTINA:** 2♂, Misiones, Aristóbulo del Valle, Valle del Cuñá Pirú, Jan 2014 (RYB); 8♂, Misiones, Iguazú, “Subtropischer Regenwald”, alt. 200 m, Sep–Oct 1996, leg. R. Foerster (MWM); 8♂, Misiones, Iguazú, “CAMP. BR.–ARG.”, 1–3 Feb 1994 (MWM); 2♂, Misiones, 18 Nov 1954, coll. Petrovsky (MLP); 1♂, Misiones, 35 km E of Puerto Bossetti, 13 Oct 2002, leg. O. Di Iorio (FCP); 1♂, Misiones, “MACN-Bar-Lep-ct 0762” (MACN); 1♂, Misiones, Aristóbulo del Valle, Camping del Valle del Cuñá Pirú, Nov 2007, coll. E. Gogliormella (EGO); 1♂, Misiones, 24 Nov 2011, leg. P. Loescher (FCP); 1♂, Misiones, 26 Sep 2012, “Prep Gen FCP №12”, leg. P. Loescher (FCP); 3♂, Misiones, 1 Jun 2014, leg. L. Aguado (LA); 1♂, Misiones, “Prep Gen FCP №5”, 1 Jun 2014, leg. L. Aguado (FCP); 2♂, Misiones, 4–5 Apr 2014, leg. L. Aguado (FCP); 5♂, Misiones, 4–7 Nov 2009, leg. L. Aguado (LA); 1♂, Misiones, Dec 2013, leg. L. Aguado (LA); 1♂, Misiones, Reserva Yacutinga, Nov 2001, leg. O. Di Iorio (FCP); 3♂, Misiones, Jun 2006, leg. O. Di Iorio (FCP); 2♂, Misiones, Parque Provincial Salto Encantado, 22 Nov 2007, coll. E. Gogliormella (EGO); 1♂, Misiones, 6–9 Nov 2009, “Prep Gen FCP №4”, leg. L. Aguado (FCP); 5♂, Misiones (LA); 1♂, Misiones, Eldorado (FML); 1♂, Misiones, Wanda, 10 Mar 1997, leg. J. Rumi, “GenPr-Heterocera MWM 26.708” (MWM); 1♂, Chaco, Depto. Chacabuco, Charata, Pozo La Gringa, 23 Jan 1996, leg. O. Di Iorio (FCP); 1♂, Buenos Aires, San Isidro, Jockey Club, 28 Dec 1991, leg. E. Nuñez Bustos (ENB).

**Diagnosis.** External morphology and male genitalia similar to its sibling species, *M. pyracmon* (Cramer, [1780]) (see Section 3.1. Phylogenetic Relationships within the Genus *Morpheis* and Section 4. Discussion). Externally both species are most similar to *M. comisteus*, *M. impeditus,* and *M. melanoleucus*. All share a light ground colour of the wings, a distinct longitudinal stripe of irregular shape on the forewings, and a pronounced wavy pattern. From *M. comisteus* the species differs in the more contrasting and darker wavy pattern, the shape of the forewing longitudinal stripe and its more defined contour relative to the wavy pattern. The species differs from *M. impeditus* in a lighter ground colour of the wings and a brownish shading of the longitudinal stripe and wavy pattern. From *M. melanoleucus* the species differs in the clearer outline of the forewing longitudinal stripe, a more uniform thickness of the streaky pattern strokes (generally thin), and a light central part of the thoracic dorsum (except for thin dark longitudinal stripes along the border with the tegulae, expressed in some specimens). In female genitalia, the species differs from *M. melanoleucus* in a smaller signum and bulla relative to the bursa and in a longer ductus bursae.

**Distribution.** Assuming that *M. putridus* and *M. pyracmon* are allopatric in their distribution (Figure 13), the range of the former encompasses the southeastern part of the Neotropical region (southeastern Brazil, Paraguay and northern Argentina), corresponding biogeographically to the Chacoan subregion (see Section 4. Discussion).

**Remarks.** Despite H.G. Dyar’s note that the species he described, *M. discretus* (=*Xyleutes discreta*), is close to *M. comisteus* (=*Xyleutes comisteon*), we consider *M. discretus* a small-sized form of *M. putridus*. This interpretation is based on shared external features, such as the dark brown forewing longitudinal stripe with a smoother outline, relatively defined contour, and uniform dark-brown tone that does not contrast with the discal spots near the outer forewing margin. As imago size in certain Zeuzerinae species interspecifically varies greatly (likely associated with larval nutritional characteristics) we do not recognise *M. discretus* as a distinct taxon. Phylogenetic analysis further confirms conspecificity between medium-sized (MORSA021-26; Figure 10A) and small-sized (MORSA022-26; Figure 10D) *M. putridus* specimens. Small-sized *M. putridus* are comparable in size to the type specimen of *X. discreta* (Figure 10E). Thus, we consider *X. discreta* a synomym of *M. putridus* (Percheron, 1838), **syn.nov.**

#### 3.2.9. *Morpheis pyracmon* (Cramer, [1780])

*Sphinx pyracmon* Cramer, [1780]: 169–170, Pl. 287, Figure B [3]

*Cossus palmarum* Herrich-Schäffer, [1853]: Figure 36 [10] [Synonymised by Dyar, 1940: 1266 [13].]

*Zeuzera fracta* Walker, 1856: 1542 [8] [Synonymised by Dyar, 1940: 1266 [13].]

*Duomitus pyracmonides* Schaus, 1901: 45 [16] [Synonymised by Dyar, 1940: 1266 [13].]

*Zeuzera lelex* Dognin, 1891: 121 [15] **syn. nov.**

Figure 10I–P, Figure 11C,D and Figure 13

**Material examined (identified by molecular data). COLOMBIA:** 1♂, Boyacá, road Otanche–Puerto Boyacá, 5.736111° N, 74.217778° W, alt. 680 m, 10–16 Feb 2016, leg. V. Sinyaev & J. Machado, “DNA preparation №37” (RYB); 1♂, Santander, road Duitama–Charalá, 6.060556° N, 73.214722° W, alt. 2000 m, 11–14 Mar 2016, leg. V. Sinyaev & J. Machado, “DNA preparation №38” (RYB); 2♂, Quindío, Valle del Cocora, E of Salento, 4.642222° N, 75.513056° W, alt. 2300 m, 11 Mar 2017, leg. V. Sinyaev, “DNA preparation №39”, “DNA preparation №53” (RYB). **ECUADOR:** 1♂, El Oro Prov., 8 km NW Piñas, 3.648056° S, 79.751111° W, alt. 900 m, 15 Apr 2012, leg. V. Sinyaev & O. Romanov, “AN MWM 14” (MWM); 1♂, Esmeraldas, 3 km E of Mompiche, 0.493611° N, 79.996111° W, 25–29 Jan 2013, leg. V. Sinyaev & O. Romanov, “AN MWM 15” (MWM). **PERU:** 1♂, Huánuco, Yuyapichis, ACP Panguana, 9.600000° S, 74.933333° W, alt. 220 m, May 2013, leg. H. Thöny, “AN MWM 13” (MWM).

**Tentatively identified as *M. pyracmon*: type material examined.** Holotype ♂ of *Zeuzera fractus*, “NHMUK 012832610” (NHMUK). Holotype ♂ of *Duomitus pyracmonides*: **MEXICO**, Orizaba, “IVe Trim. 1889”, “Collection WmSchaus”, “type No. 12567 USNM”, “USNMENT 01198208” (USNM). Holotype ♀ of *Zeuzera lelex*: **VENEZUELA**, Merida, 1890, “Dognin collection”, “type No. 30859 USNM”, “USNMENT 01198239” (USNM). **Additional material examined. MEXICO:** 2♂, Sierra Santa Martha, Veracruz, 18.200000° N, 95.532400° W, alt. 1400 m, Jul 2013, “GenPr-Heterocera MWM 36.973” (RYB); 1♂, Oaxaca, Finca Monte Carlo, 15.993333° N, 96.105000° W, alt. 890 m, 26–31 Aug 2011, leg. V. Sinyaev (RYB); 1♂, Oaxaca, Juquila, Lachao Nuevo, alt. 1040 m, 14 Jun 2010, leg. P. Schmit (RYB); 1♂, Puebla, La Joya, alt. 898 m, 6 Apr 1953, coll. F. Harting, “GenPr-Heterocera MWM 36.973” (MWM); 1♂, Oaxaca Prov., Metates, 40 km S of Valle Nacional, alt. 900 m, 5–13 Jul 1998, leg. Saik, “GenPr-Heterocera MWM 36.974” (MWM); 2♂, Chiapas, Tapachula, Finca Violeta, Jul–Aug [19]54, leg. Graf Harting (ZSM); 1♂, same locality, 15 Oct [19]54, leg. Graf Harting (ZSM); 1♂, Chiapas, Jun [19]26 (ZSM); 1♂, Puebla, Villa Juárez, alt. 1100 m, May [19]54, leg. Dr. C. Mayer (ZSM). **BELIZE:** 1♂, Upper Raspaculo Valley (ex British Honduras), 16.816667° N, 88.800000° W, alt. 445 m, at light, 21 May 1993, [leg.] Marcus Matthews, “Joint Services Scientific Exped. to the Upper Raspaculo 1993”, “*Morpheis pyracmon* ? 137–52 Cramer det. M.R. Honey 1993” (NHMUK). **EL SALVADOR:** 1♂, San Salvador, alt. 600 m, 2 Nov 1953, leg. B. Bechyně (ZSM). **NICARAGUA:** 6♂, Estelí, Miraflores, “UTM 16P-0580805-1464422”, alt. 1434 m, 26–27 Oct 2001, coll. J.M. Maes & B. Téllez (RYB); 2♂, Chontales, 2 km E of Santo Domingo, “UTM 16P-708241-1358398”, alt. 580 m, 15–17 Sep 2001, coll. J.M. Maes (RYB); 4♂, Granada, Volcán Mombacho, alt. 1150 m, 2002, coll. J.M. Maes (RYB). **COSTA RICA:** 1♂, Heredia, Cari Blanco, “L.F.”, 24 Feb [19]87, leg. Berlin Flauger (MWM); 1♂, Guanacaste, 2 km W of Arenal, 10.330000° N, 84.540000° W, alt. 600 m, “Coll. Nr. 388”, leg. Görner (MWM); 1♂, San José, 25 km SW San Isidro de El General, coastal area near Dominical, alt. 10–250 m, 19–28 Nov 1995, leg. de Freina (RYB). **PANAMA:** 3♂, Ngäbe-Buglé, La Vemigosa, Fortuna Forest Reserve, 8.780000° N, 82.191000° W, alt. 820 m, Aug–Sep 2021, leg. A. Kozlov (RYB); 1♂, Lino, alt. 800 m, coll. Fassl, “ex Joicey Coll. Brit. Mus. 1923–137” (NHMUK); 1♂, “El Valle, Panama”, 24 Nov 1946, “R.E. Ellison coll. B.M. 1960–550” (NHMUK). **COLOMBIA:** 8♂, Cundinamarca, Municipio Ubalá, Algodones, 4.697222° N, 73.370278° W, alt. 1300 m, 16–18 Dec 2017, leg. V. Sinyaev & J. Machado (RYB); 1♂, Cundinamarca, road Guaduas–Honda, 5.099444° N, 74.627778° W, alt. 1260 m, 1–2 Nov 2015, leg. V. Sinyaev & M. Márquez (MWM); 2♂, Cundinamarca, Monterredondo, alt. 1420 m, 11 Feb [19]59, leg. J. Förster (ZSM); 7♂, Meta, Municipio Restrepo, 4.291944° N, 73.594722° W, alt. 900 m, 5–8 Jan 2018, leg. V. Sinyaev & J. Machado (RYB); 1♂, Antioquia, Municipio San Luis, Vereda Filo de Ambre, 5.984167° N, 74.953056° W, alt. 770 m, 19–20 Nov 2015, leg. V. Sinyaev & M. Márquez (RYB); 1♂, Cauca, 42 km road Mocoa–Pitalito, 1.413611° N, 76.493889° W, alt. 1100 m, 15–16 Feb 2018, leg. V. Sinyaev & J. Machado (RYB); 1♂, Cesar, Sierra Nevada de Santa Marta (E), 5 km S of Pueblo Bello, Finca Paraíso, 10.349444° N, 73.566111° W, alt. 2700 m, 13–14 Jun 2018, leg. V. Sinyaev, L. da Vinci & V. Gromenko (RYB); 2♂, Quindío, Valle del Cocora, E of Salento, 4.642222° N, 75.513056° W, alt. 2300 m, 11 Mar 2017, leg. V. Sinyaev & C. Pinilla (RYB); 1♂, Huila, 25 km SW of Gigante, 2.342778° N, 75.595278° W, alt. 777 m, 4 May 2018, leg. V. Sinyaev & V. Gromenko (RYB); 2♂, Santander, road Duitama–Charalá, 6.060556° N, 73.214722° W, alt. 2000 m, 11–14 Mar 2016, leg. V. Sinyaev, V. Zaritzki & R. Gortovanyi (RYB); 1♂, Santander, rd. Barbosa–Arcabuco km 23, 5.820556° N, 73.503889° W, alt. 2360 m, 21–27 Jan 2014, leg. V. Sinyaev (MWM); 1♂, Santander, Vereda de Buenos Aires, San Juan Curí del Páramo, 6.348889° N, 73.166944° W, alt. 1520 m, 22–23 Nov 2013, leg. V. Sinyaev & M. Márquez (MWM); 1♂, Alto Río Opón, Santander del Sur, alt. 1050–1900 m, light trap, 14–26 Aug [19]48, leg. L. Richter (ZSM); 1♂, Boyacá, road Arcabuco–Togüí, 5.820833° N, 73.503889° W, alt. 2350 m, 7–8 Dec 2015, leg. V. Sinyaev & M. Márquez (RYB); 8♂, Boyacá, road Otanche–Puerto Boyacá, 5.736111° N, 74.217778° W, alt. 685 m, 10–16 Feb 2016, leg. V. Sinyaev, V. Zaritzki & R. Gortovanyi (RYB); 13♂, Magdalena, road Minca–Cerro Kennedy (W), 11.125278° N, 74.101944° W, alt. 1000 m, 7–8 Jun 2018, leg. V. Sinyaev, L. da Vinci & V. Gromenko (RYB); 1♂, Magdalena, Municipio de Minca, near Minca, 11.125278° N, 74.101944° W, alt. 1100 m, 17–18 Jul 2017, leg. V. Sinyaev & C. Pinilla (RYB); 2♂, 1♀, Magdalena, Municipio de Minca, Reserva El Faunal, 11.132222° N, 74.102778° W, alt. 800 m, 19–21 Nov 2014, leg. V. Sinyaev & M. Márquez (MWM); 2♂, Magdalena, Municipio de Minca, Vereda Bella Vista, Hostal Palo Alto, 11.096389° N, 74.076111° W, alt. 1680 m, 24–28 Nov 2014, leg. V. Sinyaev & M. Márquez (MWM); 1♂, Antioquia, Municipio de Cocorná, Vereda Borojo, 6.020278° N, 75.125556° W, alt. 1080 m, 22–23 Nov 2015, leg. V. Sinyaev & M. Márquez (MWM); 1♂, “Casoba”, 1961, coll. Marga & Bos (FML); 1♂, Río Magdalena, “Bodillo”, 17 Apr [1]926, [leg.] Woronov (ZISP); 1♂, Río Magdalena, Peñas Blancas, 26 Apr–5 May [1]926 (ZISP); 1♂, Chocó, El Tigre, Río Tamaus, alt. 820 ft, Feb [19]09, [leg.] G.M. Palmer, “Ex Coll. H. Druce 1913”, “Joicey Coll. Brit. Mus. 1925–157” (NHMUK); 1♂, Río Magdalena, Barranquilla to El Banco, Jun–Jul 1924, [leg.] C. Allen, “Brit. Mus. 1924–407” (NHMUK); 1♂, Muzo, R. Cantineiro, alt. 400 m, [leg.] A.H. Fassl (NHMUK); 1♂, Magdalena, Sierra Nevada de Santa Marta, alt. 1400 m, 10.900000° N, 74.050000° W, “Oxford Expedn to Colombia.”, 3 Jul–2 Sep 1973, “B.M.1973–500” (NHMUK); 1♂, Chocó, Tadó, Río San Juan, alt. 250 ft, Jun 1909, leg. G.M. Palmer (Ex Coll. Herbert Druce, 1913) (NHMUK); 1♂, Magdalena Valley, May–Aug 1920, [leg.] A. Hall, “Joicey Coll. Brit. Mus. 1925–157” (NHMUK); 1♂, “Colombia” (NHMUK); 3♂, Río Magdalena, 23 Mar 1947, leg. H. Lunde (NHM-UiO). **VENEZUELA:** 1♂, Yaracuy, Cordillera de la Costa, 10.248333° N, 68.518333° W, alt. 780 m, 16 Aug 2007, leg. Th. Greifenstein (MWM); 5♂, Carabobo, Cordillera de la Costa, Bejuma, “Casa María”, alt. 500–1600 m, 13 Aug–2 Sep 2007, leg. Th. Greifenstein (MWM); 1♂, Bolívar, Gran Sabana, Sierra Pacaraima, Río Surukun, 4.610000° N, 61.429500° W, alt. 950 m, 21–23 Aug 2008, leg. Th. Greifenstein (MWM); 3♂, Maracay, coll. P. Vogl, “*Xyleutes pyracmon* Cr. det. W. Förster München” (ZSM); 3♂, same locality, Nov–Dec 1934 (ZSM); 1♂, same locality, Aug 1934 (ZSM); 1♂, same locality, Jul 1934 (ZSM); 1♂, same locality, May 1934 (ZSM); 1♂, Aragua, Rancho Grande, 12 Jul–16 Aug 1976, [leg.] A. Watson, “B.M. 1976–552” (NHMUK); 1♂, Auyán Tepui, Camarata, 4–17 Aug 1974, [leg.] B.V. Ridout, “B.M. 1974–650” (NHMUK); 1♂, San Esteban, Jun 1909, [leg.] S.M. Klages (NHMUK); 1♂, Mérida, “(Briceño)” (NHMUK); 1♂, Mérida, “R. 100”, “Meyrick Coll. B.M. 1988–290” (NHMUK); 1♂, Valencia, “Doncaster private coll., purch. 1927” (OUMNH). **TRINIDAD AND TOBAGO:** 1♂, Trinidad, Caparo, Dec 1905, [leg.] S.M. Klages (NHMUK). **GUYANA:** 1♂, “bought at Georgetown by Mr. Whitford” (NHMUK); 1♂, Omai, Jun 1908, [leg.] S.M. Klages (NHMUK). **SURINAME:** 1♂, Aroewarwa Creek, Marowijne Valley, Jul 1905, leg. S.M. Klages, “SLIDE NHMUK 014333367”, “NHMUK 010290890” (NHMUK). **FRENCH GUIANA:** 3♂, Piste de Kaw, Aug 2000, leg. B. Penin (RYB); 1♂, St. Jean de Maroni, “Received from R. Le Moult” (NHMUK). **BRAZIL:** 1♂, Rondônia, vicinity of Cacaulândia, Rancho Grande, alt. 350 m, Nov 1999, leg. R. Alves dos Santos (MWM); 1♂, Upper Amazon, Santo Antonio do Javary, May [19]07, [leg.] S.M. Klages (NHMUK); 1♂, “500 m up R. Madeira and 50 miles east”, V. Cressy-Marcks, “B.M. 1931–594” (NHMUK); 2♂, Upper Amazon, Codajás, Mar–Apr 1907, [leg.] S.M. Klages (NHMUK); 1♂, Amazonas, Fonte Boa, Jul 1906, [leg.] S.M. Klages (NHMUK). **ECUADOR:** 1♂, El Oro Prov., 8 km NW Piñas, 3.648056° S, 79.751111° W, alt. 900 m, 15 Apr 2012, leg. V. Sinyaev & O. Romanov (MWM); 1♂, Esmeraldas, 3 km E of Mompiche, 0.493611° N, 79.996111° W, 25–29 Jan 2013, leg. V. Sinyaev & O. Romanov (MWM); 1♂, Imbabura, Ibarra District, alt. 2700 m, 17 Feb 2012, leg. A. Zorin (MWM); 5♂, Esmeraldas, 25.83 km N of Lita, 1.031089° N, 78.614664° W, alt. 349 m, 21 Nov 2021, leg. V. Doroshkin & R. Gortovannyi (RYB); 1♂, Pichincha, Río Pachijal, Los Bancos, 0.074167° N, 78.908611° W, alt. 700 m, 15 Dec 2012, leg. V. Sinyaev & O. Romanov (MWM); 4♂, Pichincha, 16 km E of Santo Domingo, alt. 650 m, 24–25 Dec 1975, leg. Mühle (MWM); 1♂, Morona Santiago, Riobamba–Macas, 2.213889° S, 78.138889° W, alt. 1200 m, 29 Mar 2012, leg. V. Sinyaev & O. Romanov (MWM); 1♂, Morona Santiago, road Puyo–Macas, 1.930556° S, 77.830000° W, alt. 700 m, 6 Mar 2013, leg. V. Sinyaev & O. Romanov (MWM); 2♂, Esmeraldas, 3.2 km E of Mompiche, 0.493611° N, 79.996111° W, alt. 140 m, 25 Apr 2012, leg. V. Sinyaev & O. Romanov (MWM). **PERU:** 2♂, Piura, Abra Porcuya, alt. 1800 m, May 2007, ex coll. Dr. R. Brechlin, “GenPr-Heterocera MWM 36.878” (MWM); 2♂, Huánuco, Yuyapichis, ACP Panguana, 9.600000° S, 74.933333° W, alt. 220 m, May 2013, leg. H. Thöny (MWM); 16♂, same locality, Jun–Aug 2013, coll. Dr. R. Brechlin (MWM); 1♂, Huánuco, Tingo María, Santa Rosa, alt. 810 m, 17 Sep 2003, “P03-02-17”, leg. Pierre Schmit (RYB); 1♂, Huánuco, Santa Rosa, environs of Tingo María, 17 Sep 2003, leg. Pierre Schmit, “P03-02-17” (RYB); 1♂, Huánuco, Tingo María, Jul 1974, leg. C. Porter & L. Stange (FML); 1♂, San Martín, 6.618190° S, 76.181080° W, alt. 260 m, Jul 2017, leg. A. Kozlov & Yu. Kovaleva (RYB); 2♂, San Martín, Mina de Sal, alt. 1400 m, 2007, leg. R. Marx (MWM); 1♂, San Martín, Rioja, alt. 900 m, May 2007, leg. R. Marx (MWM); 1♂, Cusco, San Miguel, Vallée Marcapata, 15 Oct 2004, leg. Pierre Schmit, “P03-11-15” (RYB); 6♂, Cusco, Chontachaca, Manu Park, alt. 800 m, Jan 1999, via R. Marx, ex coll. A. Shintlmeister (MWM); 1♂, Cusco, Valle de Quillabamba, alt. 1800 m, Oct–Nov 2006, leg. R. Marx (MWM); 3♂, Amazonas, El Paraíso, alt. 2400 m, Oct–Nov 2006, leg. R. Marx (MWM); 1♂, Amazonas, Utcubamba, Puente Nieva, 4.833333° S, 77.950000° W, alt. 800 m, Dec 2007, coll. Dr. R. Brechlin (MWM); 5♂, Amazonas, Puente Nieva, alt. 700–800 m, Jun 2007, coll. R. Marx (MWM); 2♂, Amazonas, Utcubamba, Montenegro, alt. 900 m, Dec 2007, coll. Dr. R. Brechlin (MWM); 24♂, Madre de Dios, Route Salvación–Pillahuata, Manu Park, alt. 600–2500 m, Oct–Nov 1998, via R. Marx, ex coll. A. Shintlmeister (MWM); 9♂, Madre de Dios, Camicana–Chico, Río Carbon, Manu Park, alt. 800–1000 m, 3 Apr 1998 (MWM); 1♂, same locality, alt. 1200 m, Jul–Aug 1997, leg. R. Marx, “GenPr-Heterocera MWM 28.483” (MWM); 3♂, same locality, Nov–Dec 1998, via R. Marx, ex coll. A. Shintlmeister (MWM); 1♂, Junín, Bajo Capiro, Satipo, alt. 900 m, 3 Apr 1997 (MWM); 1♂, Junín, Satipo, Shanki, alt. 700 m, 4–15 Aug 1997, leg. A. Ugarte (MWM); 1♂, Iquitos (RMNH); 1♂, Río Huallaga, alt. 670 m, Oct 1947, leg. Weyrauch (FML); 1♂, Carabaya, La Unión, R. Huacamayo, alt. 2000 ft, “wet s.”, Dec 1904, [leg.] G. Ockenden (NHMUK); 1♂, Yahuarmayo, alt. 1200 ft, May–Jul 1912, leg. Watkins (Ex Coll. Herbert Druce, 1913) (NHMUK); 1♂, “La Merced”, “C. Peru”, 3000–4500 ft, Jan–Feb 1920, C. Watkins, “Joicey Coll. Brit. Mus. 1925–157” (NHMUK). **BOLIVIA:** 2♂, La Paz, Incahuara, alt. 800 m, 30 Oct 2004, leg. P. Schmit (RYB); 1♂, Santa Cruz, 8.3 km W of Mataral, 18.128333° S, 64.285000° W, alt. 1661 m, 19 Jan 2010, leg. V. Sinyaev, S. & A. Zamesov (RYB); 1♂, Santa Cruz, Santiago de Chiquitos, Nov 2009, leg. L. Aguado (LA); 1♂, Chapare, San Jacinto, alt. 2800 m, 15 Nov 1996, leg. Lf. Ugarte (MWM); 3♂, Chapare, upper Río Chipiriri area, alt. 400 m, 25 Oct 1953, leg. W. Förster (ZSM); 7♂, Chapare, 400 m, Oct [19]53, leg. R. Zischka (ZSM); 1♂, same locality, 5 Oct [19]50, leg. R. Zischka (ZSM); 1♂, same locality, 10 Aug [19]50, leg. R. Zischka (ZSM); 1♂, same locality, 1 Oct [19]50, leg. R. Zischka (ZSM); 1♂, same locality, 5 Dec [19]50, leg. R. Zischka (ZSM); 1♂, same locality, 5 Nov [19]51, leg. R. Zischka (ZSM); 1♂, same locality, 23 Apr [19]48, leg. R. Zischka (ZSM); 1♂, same locality, 15 Apr [19]48, leg. R. Zischka (ZSM); 2♂, same locality, 20 Apr [19]49, leg. R. Zischka (ZSM); 3♂, Amazonica, Trinidad, Oct [19]51 (ZSM).

**Diagnosis.** External morphology and male genitalia similar to its sibling species, *M. putridus* (see Section 3.1. Phylogenetic Relationships within the Genus *Morpheis* and Section 4. Discussion). Externally both species are most similar to *M. comisteus*, *M. impeditus* and *M. melanoleucus* (see Section 3.2.8. Diagnosis of *M. putridus*). The female genitalia are unknown.

**Distribution.** Assuming that *M. putridus* and *M. pyracmon* are allopatric in their distribution (Figure 13), the range of the latter encompasses northwestern part of the Neotropical region (southern Mexico, Central America, French Guiana, Suriname, Guyana, Venezuela, Trinidad and Tobago, Colombia, Ecuador, Peru, Bolivia, and northwestern Brazil), corresponding biogeographically to the Brazilian subregion (see Section 4. Discussion).

**Remarks.** The holotype of *Zeuzera lelex* Dognin, 1891 from USNM (Figure 10O) corresponds morphologically to a typical female of the cryptic species *M. putridus* and *M. pyracmon* (Figure 10P). Considering the type locality of *Z. lelex* (Venezuela, Merida), which falls within the allopatric range of *M. pyracmon* we can assume that this specimen belongs to *M. pyracmon.* Thus, we consider *Z. lelex* Dognin, 1891 a synonym of *M. pyracmon* (Cramer, [1780]), **syn. nov.**

#### 3.2.10. *Morpheis votani* (Schaus, 1934)

*Xyleutes votani* Schaus, 1934: 95 [24]

**Figure 14:**
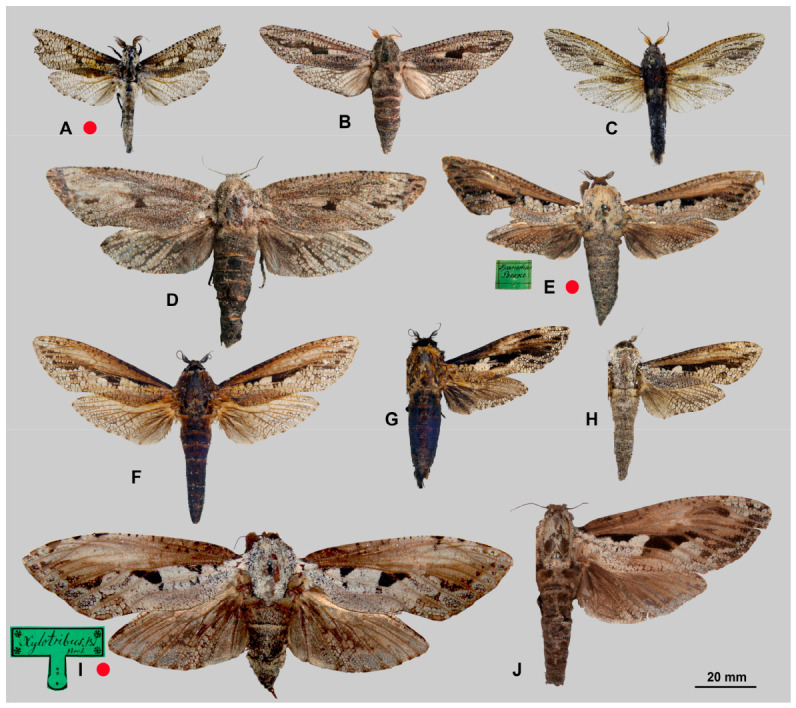
*Morpheis* imagoes: (**A**) *M. votani* (Schaus, 1934), male, holotype, Argentina, La Rioja (USNM); (**B**) *M. votani* (Schaus, 1934), male, Argentina, La Rioja (MHNH); (**C**) *M. votani* (Schaus, 1934), male, Paraguay, Presidente Hayes (RYB); (**D**) *M. votani* (Schaus, 1934), female, Argentina, La Rioja (MHNH); (**E**) *M. xylotribus* (Herrich-Schäffer, [1853]), male, lectotype, [Brazil] (ZISP); (**F**) *M. xylotribus* (Herrich-Schäffer, [1853]), male, Brazil, Bahia (MWM); (**G**) *M. xylotribus* (Herrich-Schäffer, [1853]), male, Columbia, Boyacá, (MWM); (**H**) *M. xylotribus* (Herrich-Schäffer, [1853]), male, Paraguay, Boquerón (MWM); (**I**) *M. xylotribus* (Herrich-Schäffer, [1853]), female, paralectotype, [Brazil] (ZISP); (**J**) *M. xylotribus* (Herrich-Schäffer, [1853]), female, Venezuela, Maracay (ZSM); red circles mark type specimens.

**Figure 15:**
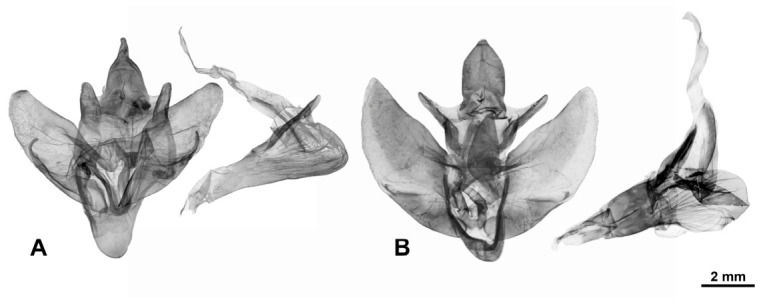
*Morpheis* male genitalia: (**A**) *M. votani* (Schaus, 1934), Paraguay, Presidente Hayes, genital slide No. 909 (RYB); (**B**) *M. xylotribus* (Herrich-Schäffer, [1853]), Argentina, Misiones, genital slide No. 36.983 (MWM).

**Figure 16:**
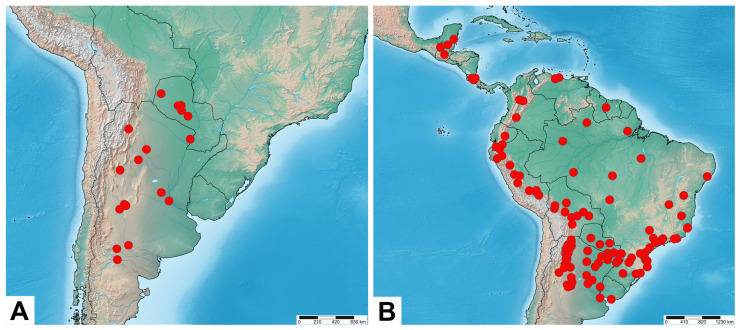
Distributional records of *Morpheis*: (**A**) *M. votani* (Schaus, 1934); (**B**) *M. xylotribus* (Herrich-Schäffer, [1853]).

Figure 14A–D, Figure 15A and Figure 16A

**Type material examined.** Holotype ♂: **ARGENTINA**, La Rioja, coll. Giacomelli, “Type No. 34519” (USNM). **Additional material examined. ARGENTINA:** 2♂, La Rioja, 1918, leg. Eugenio Giacomelli (MHNH); 1♂, 1♀, same locality, coll. R. Biedermann (MHNH); 2♂, La Rioja (MLP); 4♂, 2♀, La Rioja, leg. Giacomelli (MACN); 1♂, Santiago del Estero, Depto. Choya, Laprida, 12 Dec 2009, leg. A. Borquez, “PG FCP No. 6” (FCP); 1♂, Santiago del Estero, Depto. Choya, Laprida, 23 Feb 2018, leg. A. Borquez, “ANRYB 143” (RYB); 1♀, Salta, Rosario de Lerma, 18 Aug 1993, leg. O. Di Iorio (FCP); 1♀, San Luis, San Gerónimo, Nov 1972, leg. Williner (MACN); 1♂, San Luis, 10 Feb 1972, leg. Williner, ex coll. O. Di Iorio (FCP); 1♂, San Luis, Jan 1972, leg. Viana (MACN); 1♀, San Luis, Nov 1992, leg. Williner, ex coll. O. Di Iorio (FCP); 1♂, Formosa, “MACN-Bar-Lep-ct 4026” (MACN). **PARAGUAY:** 1♂, Presidente Hayes Dept., Estancia Salazar, 23.066667° S, 59.250000° W, 17–18 Nov 2012, leg. U. Drechsel, “ANRYB 139” (RYB); 3♂, Presidente Hayes Dept., Chaco Lodge, 22.500000° S, 59.300000° W, 4–6 Nov 2015, leg. U. Drechsel (RYB); 1♂, same locality, 22–25 Oct 2015, leg. U. Drechsel (RYB); 2♂, Presidente Hayes Dept., Costa Esmeralda, 23.666667° S, 58.483333° W, 18 Jan 2011, leg. U. Drechsel (RYB); 1♂, Presidente Hayes Dept., Laguna Capitán, 22.533333° S, 59.666667° W, 12–14 Sep 2017, leg. U. Drechsel (RYB); 1♂, Boquerón Dept., Enciso, 21.200000° S, 61.650000° W, 6–11 Jan 2015, leg. U. Drechsel (RYB).

**Diagnosis.** Externally, the species is most similar to *M. impeditus*. Both species share a grey ground colour of the wings. However, *M. votani* is distinctive in habitus and exhibits clear differences in male genitalia from all other species. In the forewing pattern, the species differs from other members of the genus by the absence of the basal part of the dark longitudinal stripe (present and adjacent to the costal margin in other species), a dense weaving of the wavy pattern, and a dark grey, speckled ground colour, most pronounced in the lower basal part of the forewing (formed by a dense mixture of grey and blackish scales). The hindwings exhibit a dark semitransparent wavy pattern on a light ground colour. The thorax and abdomen are dark grey, speckled due to the alternation of dark and grey hairs. In male genitalia, the apex of the uncus is narrowed; the harpe on the valves is clearly expressed, curved, and sclerotised; the saccus is robust, elongated; the lateral processes of the juxta bear spherical laterally directed proliferations; the carina of the aedeagus with numerous fine teeth on its surface.

**Distribution.** Paraguay and Argentina (south to the Monte Desert) (Figure 16A).

#### 3.2.11. *Morpheis xylotribus* (Herrich-Schäffer, [1853])

*Cossus xylotribus* Herrich-Schäffer, [1853]: Pl. 8, Figures 37 and 38 [10]

**Figure 17:**
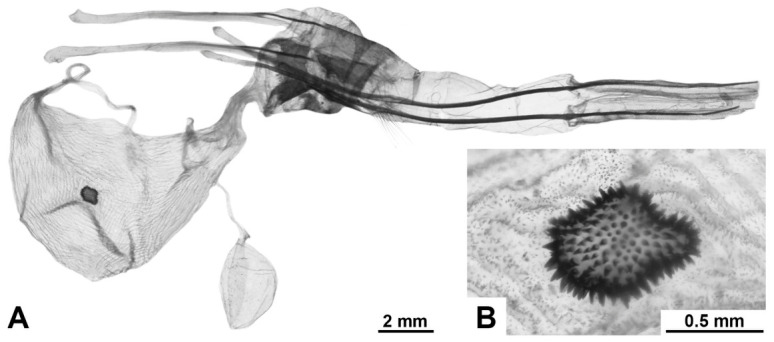
Female genitalia of *Morpheis xylotribus* (Herrich-Schäffer, [1853]), Venezuela, Maracay, genitalia slide No. 1443 (MWM): (**A**) general view; (**B**) signum, enlarged.

Figure 14E–J, Figure 15B, Figure 16B and Figure 17

**Type material examined.** Lectotype ♂ (here designated), [Brazil], “Ipanema / Beske” (ZISP). Paralectotype 1♀ (here designated), [Brazil] (ZISP). **Additional material examined. MEXICO:** 3♂, Estado Chiapas, Lacanjá, 135 km SW Palenque, alt. 320 m, 7 Jun 2010, leg. P. Schmit (RYB). **BELIZE:** 2♂, Corozal (ex Brit. Honduras), [leg.] F.L. Davis, “B.M. 1926–399” (NHMUK). **GUATEMALA:** 1♂, Baja Verapaz, Purulhá, Finca Santa Rosa, primary *Quercus* mixed forest, 15.238333° N, 90.285000° W, alt. 1650 m, leg. J.P. Rudloff, “G2” (MWM). **COLOMBIA:** 1♂, Boyacá, road Otanche–Puerto Boyacá, 5.736111° N, 74.217778° W, alt. 680 m, 18–20 Mar 2016, leg. V. Sinyaev & J. Machado, “GenPr-Heterocera MWM 36.979”, “AN MWM 5” (MWM); 1♂, Huila, 25 km SW Gigante, 2.342778° N, 75.595278° W, alt. 777 m, 4 May 2018, leg. V. Sinyaev & V. Gromenko (RYB); 3♂, Antioquia, Municipio San Luis, Vereda Filo de Ambre, 5.984167° N, 74.953056° W, alt. 770 m, 19–20 Nov 2015, leg. V. Sinyaev, J. Machado & R. Brechlin (MWM). **VENEZUELA:** 1♀, Maracay, Jul [19]34, leg. P. Vogl (ZSM); 2♀, same locality, leg. P. Vogl (ZSM); 5♂, same locality, “ges. P. Vogl” (ZSM); 2♂, same locality, May [19]34, leg. P. Vogl (ZSM); 1♂, same locality, Aug [19]34, leg. P. Vogl (ZSM); 1♀, Caracas, Cerro Ávila, 11–14 Aug [19]36, “P. Cor. Vogl” (ZSM); 1♂, same locality, 10–19 Apr [19]26, “P. Cor. Vogl” (ZSM); 1♂, Estado Aragua, Rancho Grande, road Maracay–Ocumare km 22, primary forest, 21 May 1947, leg. R. Lichy, “Carnegie Mus. Acc. 18484” (CMNH). **ECUADOR:** 1♂, Pastaza, 9.7 km NE Puyo, 1.457500° S, 77.919722° W, alt. 1066 m, leg. R. Brechlin & V. Sinyaev (MWM); 2♂, El Oro, 8 km NW Piñas, 3.648056° S, 79.751111° W, alt. 900 m, 15 Apr 2012, leg. R. Brechlin & V. Sinyaev (MWM); 2♂, Morona-Santiago Prov., San Carlos de Limón, 3.183889° S, 78.444444° W, alt. 937 m, 24 Nov 2011, leg. V. Sinyaev & O. Romanov, “AN MWM 3” (MWM); 1♂, same data, “DNA preparation №85” (RYB). **PERU:** 19♂, Huánuco, Yuyapichis, ACP Panguana, 9.600000° S, 74.933333° W, alt. 220 m, Jun–Jul 2013, leg. H. Thöny, “coll. Dr. R. Brechlin”, “GenPr Heterocera MWM 36.980”, “AN MWM 1” (MWM); 1♂, same locality, May 2013, leg. H. Thöny (MWM); 2♂, Amazonas, Utcubamba, Montenegro, alt. 900 m, Dec 2007, coll. Dr. R. Brechlin, “AN MWM 2” (MWM); 2♂, same locality, alt. 800 m, Dec 2007, coll. Dr. R. Brechlin (MWM); 1♂, Madre de Dios, Salvación, Río Alto Madre de Dios, Manu Park, alt. 500 m, Oct–Nov 1996 (RYB); 8♂, same locality, alt. 400 m, Oct–Nov 1998, “via R. Marx, ex coll. Shintlmeister” (MWM); 5♂, same locality, alt. 600 m, Nov–Dec 1998 (MWM); 1♂, Madre de Dios, Camicana–Chico, Río Carbón, Manu Park, alt. 800–1000 m, Mar–Apr 1998 (MWM); 1♂, Cusco, Pont. Capiri, Valle Marcapata, alt. 1300 m, 14 Oct 2004, “P044-09-14”, leg. P. Schmit (RYB); 1♂, Cusco, Río Unebamba, Koribeni, alt. 700 m, 1–5 Dec 1994, leg. Hácz & Juhánz (MWM); 1♂, Lambayeque Dept., Motupe Distr., Colaya, 6.083333° S, 79.516667° W, alt. 1800 m, Dec 2007, ex coll. Dr. R. Brechlin (MWM); 1♂, Rentema Falls, Upper Marañón, alt. 1000 ft, [leg.] A. & E. Pratt (NHMUK); 1♂, La Oroya, Río Inambari, alt. 3100 ft, dry season, Sep 1904, leg. G. Ockenden (NHMUK); 1♂, La Merced, alt. 3000–4500 ft, Jan–Feb 1920, [leg.] C. Watkins, “Joicey Coll. Brit. Mus. 1925–157” (NHMUK); 3♂, Carabaya, La Unión, Río Huacamayo, 2000 ft, Nov–Dec 1904, [leg.] G. Ockenden, “Joicey Coll. Brit. Mus. 1925–157”, “Ex Coll. Herbert Druce 1913” (NHMUK); 1♂, Tingo María, Río Huallaga, alt. 570 m, leg. Weyrauch (FML). **BOLIVIA:** 1♂, Santa Cruz, San José de Chiquitos, Chiquitos N.P., alt. 550 m, May/Jun 2009, “leg. local collector, coll. Dr. Ronald Brechlin”, “GenPr-Heterocera MWM 36.981” (MWM); 1♂, 29.5 km SE Padilla, 19.531667° S, 64.161667° W, alt. 1545 m, leg. V. & S. Sinyaev, A. Zamesov (MWM); 3♂, 8.3 km W Mataral, 18.128333° S, 64.285000° W, alt. 1661 m, leg. V. & S. Sinyaev, A. Zamesov (RYB); 1♂, La Paz, “Death Road” (Coroico), 16.305000° S, 67.813333° W, alt. 3060 m, leg. V. Sinyaev & O. Romanov (RYB); 2♂, Cochabamba, Chocolatal, May 2017, leg. A. Borquez (RYB); 1♂, Santa Cruz, alt. 500 m, 3 Dec [19]55, leg. R. Eischka (ZSM); 1♂, Cristal Mayú, Jan 2015, leg. Calderón & Borquez (FCP); 1♂, Río Songo, alt. 750 m, leg. Fassl, “Joicey Coll. Brit. Mus. 1923–157” (NHMUK); 1♂, Chuquisaca, Feb 2015, leg. A. Borquez (FCP). **BRAZIL:** 1♂, Bahia “P.N.”, Chapada, Lençóis, alt. 400 m, Jan–Feb 1998, leg. H. Thöny, “GenPr-Heterocera MWM 36.982” (MWM); 2♂, Minas Gerais, Rte Braseipolis, Conceição d’Ouros, alt. 920 m, 18 Jan 2004, “B04-05-18”, leg. P. Schmit (RYB); 1♂, Minas Gerais, d’Ouros, alt. 880 m, 21 Jan 2004, “B04-08-21”, leg. P. Schmit (RYB); 28♂, Minas Gerais, Pote, alt. 500 m, 15 Sep–15 Oct 1996, leg. H. Thöny (MWM); 1♂, same locality, 20 Feb 1996, leg. H. Thöny (MWM); 1♂, same locality, 15 Apr 1996, leg. H. Thöny (MWM); 1♂, same locality, 15 Jul 1996, leg. H. Thöny (MWM); 1♂, same locality, 30 Jul 1996, leg. H. Thöny (MWM); 1♂, same locality, alt. 300 m, 20 Apr 1994, leg. H. Thöny (MWM); 2♂, same locality, 20 Mar 1994, leg. H. Thöny (MWM); 26♂, Minas Gerais, alt. 300 m, 12–30 Nov 1995, leg. Greifensten (MWM); 2♂, same locality, 1 Mar 1996, leg. H. Thöny (MWM); 1♂, same locality, 15 Mar 1996, leg. H. Thöny (MWM); 1♂, same locality, 15 May 1996, leg. H. Thöny (MWM); 1♂, same locality, 30 May 1996, leg. H. Thöny (MWM); 1♂, same locality, 15 Jun 1996, leg. H. Thöny (MWM); 5♂, Espírito Santo, Santa Leopoldina, Dorf Tirol, alt. 700 m, 20 Feb–30 Mar 1997, leg. H. Thöny (MWM); 1♂, same locality, 20 Mar–20 Apr 1997, leg. H. Thöny (MWM); 4♂, same locality, 15 Mar–20 Apr 1997, leg. H. Thöny (MWM); 3♂, same locality, 15 Jun 1996, leg. H. Thöny (MWM); 25♂, same locality, 22–31 Oct 1996, leg. H. Thöny (MWM); 2♂, same locality, 10–25 Nov 1996, leg. H. Thöny (MWM); 3♂, Espírito Santo, Santa Leopoldina, Boquerão, alt. 600 m, 15 Sep 1997, leg. H. Thöny (MWM); 1♀, Espírito Santo, Santa Leopoldina, Biriricas, alt. 700 m, Sep 1998, leg. H. Thöny (MWM); 2♂, Mato Grosso, Alto Paraguai, alt. 300 m, Jan 1997, leg. H. Thöny (MWM); 1♂, Barra da Estiva, Jussiape, alt. 650 m, 10–20 Apr 1997 (MWM); 1♂, Santa Catarina, S. Bento do Sul, Serra Rio Natal, alt. 850 m, Apr 1999, leg. H. Thöny (MWM); 1♂, Santa Catarina, “Nova Teutonia”, 28 Oct 1951 (MWM); 3♂, Rio Grande do Sul, Hamburgo Velho, [leg.] C. Eitl (ZSM); 1♂, Blumenau, 1930, leg. Schaus (det. as “putridus”) (FML); 1♂, Upper Amazon, Fonte Boa, Aug 1907, [leg.] S.M. Klages (NHMUK); 1♂, Aliança, below Santo Antônio, Rio Madeira, Nov–Dec 1907, [leg.] W. Hoffmanns (NHMUK); 1♂, “Brazil” (OUMNH). **PARAGUAY:** 1♂, Presidente Hayes, Costa Esmeralda, 23.666667° S, 58.483333° W, 10–14 Nov 2013, leg. U. Drechsel (RYB); 1♂, Boquerón, Toledo, 22.350000° S, 60.333333° W, 25–27 Feb 2017, leg. U. Drechsel, “DNA preparation №84” (RYB); 1♂, Cordillera, Piraretã, 25.483333° S, 56.933333° W, 13–18 Feb 2012, leg. U. Drechsel, “DNA preparation №9” (RYB); 1♂, Villarrica, Nov 1944 (MLP); 1♀, Colonia Independencia, 10 May 1951 (MLP); 5♂, Río Ypané, Cororó, 19[??], leg. Viana (MACN). **ARGENTINA:** 1♂, Jujuy, Villamonte, Portal de Piedra, Jan 2011, leg. L. Aguado (RYB); 1♂, Jujuy, 10 km SEE Caimancito, 23.744028° S, 64.519083° W, alt. 371 m, 5 Nov 2019, leg. R.V. Yakovlev (RYB); 4♂, Jujuy, 30 km NNE San Pedro de Jujuy, 24.087806° S, 64.813056° W, alt. 505 m, 27 Oct 2019, leg. R.V. Yakovlev (RYB); 2♂, Jujuy, 10 km SEE Caimancito, 23.744056° S, 64.519222° W, alt. 405 m, 25–26 Oct 2019, leg. R.V. Yakovlev (RYB); 17♂, Jujuy, Santa Bárbara Mts., 12 km SW Palma Sola, Eco Portal de Piedra NP, 24.095167° S, 64.399139° W, alt. 1045 m, 29 Oct–2 Nov 2019, leg. R.V. Yakovlev (RYB); 3♂, same mountains, 12 km SW Palma Sola, Eco Portal de Piedra NP, 24.095139° S, 64.399111° W, alt. 1045 m, 19–22 Oct 2019, leg. R.V. Yakovlev (RYB); 5♂, Jujuy, Palma Sola, 12 Oct 2015, leg. A. Borquez, ex coll. F.C. Penco (RYB); 1♂, Jujuy, Las Lancitas, 13 Jan 2016, leg. A. Borquez, ex coll. F.C. Penco, “DNA preparation №87” (RYB); 2♂, Jujuy, Rte PN Calilegua–Valle Grande km 7, alt. 1580 m, 23 Nov 2003, leg. P. Schmit (RYB); 4♂, Jujuy, Route Santa Clara–Palma Sola km 17, alt. 1180 m, 20 Nov 2003, leg. P. Schmit (RYB); 2♂, Jujuy, Villamonte, Portal de Piedra, 23 Jan 2009, leg. A. Borquez (RYB); 1♂, Jujuy, Palma Sola, 25 Jan 2009, leg. A. Borquez (RYB); 3♂, Jujuy, 23–24 Jan 2009, leg. A. Borquez (FCP); 1♂, Jujuy, Camino a El Talar, 12 Dec 2006, leg. F. Navarro (FML); 1♂, Jujuy, Depto. Santa Bárbara, Villamonte, Las Lancitas, Jan 2011, leg. L. Aguado, “Prep Gen FCP No. 10” (FCP); 3♂, Jujuy (LA); 2♂, Jujuy, 13 Jan 2016, leg. A. Borquez (FCP); 1♂, Jujuy, Palma Sola, 12 Oct 2015, leg. A. Borquez (FCP); 1♂, Jujuy, Depto. Ledesma, Caimancito, alt. 300 m, 26 Jan 2009, “0519”, leg. L. Aguado, “Prep Gen FCP No. 2” (FCP); 6♂, Salta, Route La Vica–Pampa Grande km 26, alt. 1600 m, 15 Nov 2003, leg. P. Schmit (RYB); 1♂, same locality, 26 Nov 2003, leg. P. Schmit (RYB); 3♂, Salta, Parque Nac. El Rey, Campo Santa Clara, alt. 936 m, 18 Nov 2003, leg. P. Schmit (RYB); 3♂, Salta, San Lorenzo, alt. 1570 m, 16 Nov 2003, leg. P. Schmit (RYB); 2♂, Salta, Parque Nac. El Rey, Chemin Pozo Verde, alt. 970 m, 17 Nov 2003, leg. P. Schmit (RYB); 1♀, Salta, coll. Breyer A. (MLP); 1♂, Salta, Orán, Vespucio, Nov 1953 (MACN); 2♂, Salta, Aguas Blancas, Jan 1999 (ENB); 2♂, Salta, 29 Nov 1948, leg. Ing. Monros (FML); 1♂, Salta, Río Bermejo, RN50 km 85, 28 Mar 1998, leg. J. Carreras (FCP); 2♂, Salta, Santa Victoria, 15 Nov 1960, leg. A. Bachmann (MACN); 2♂, Salta, Angosto del Río Pescado, 18 Nov 1985, leg. F. Navarro (FML); 1♂, Salta, Arrayanes, 7 Dec 1984, leg. F. Navarro (FML); 1♂, Salta, Finca El Arrozallal, 15 Nov 1985, leg. F. Navarro (FML); 2♂, Santiago del Estero, Laprida, Feb 2018, leg. A. Borquez, ex coll. F.C. Penco (RYB); 2♂, Santiago del Estero, Printo, Feb 2018, leg. A. Borquez, ex coll. F.C. Penco (RYB); 1♂, Santiago del Estero, Laprida, 29 Jan 2009, leg. A. Borquez (FCP); 2♂, 3♀, Misiones, Iguazú, “Subtropischer Regenwald”, alt. 200 m, Sep–Oct 1996, leg. R. Foerster, “GenPr-Heterocera MWM 36.983” (MWM); 1♂, Misiones, Depto. Cainguás, Camping del Valle del Cuñá Pirú, 4–7 Nov 2009, leg. L. Aguado (RYB); 1♂, Misiones, Aristóbulo del Valle, Jan 2014, leg. L. Aguado (RYB); 1♂, Misiones, Aristóbulo del Valle, Camping del Valle del Cuñá Pirú, 20–26 Oct 2019, leg. P. Loescher (RYB); 1♂, Misiones, Oberá, Villa Bonita, 15–16 Nov 2017, leg. L. Aguado, ex coll. F.C. Penco, “DNA preparation №86” (RYB); 1♂, Misiones, “MACN-Bar-Lep-ct 0945” (MACN); 1♂, Misiones, “MACN-Bar-Lep-ct 0520”, 14 Dec 2010 (MACN); 1♂, Misiones, Depto. Gral. Manuel Belgrano, Andresito, Reserva Yacutinga, Dec 2006, leg. O. Di Iorio (FCP); 1♂, Misiones, Depto. Cainguás, PP Salto Encantado, 6–9 Nov 2009, leg. L. Aguado, “Prep Gen FCP No. 1” (FCP); 1♂, Misiones, Aristóbulo del Valle, Valle del Cuñá Pirú, 20 Nov 2008, leg. L. Aguado, “Prep Gen FCP No. 9” (FCP); 1♂, Misiones, Aristóbulo del Valle, 1–13 Jan 2014, leg. F.C. Penco (FCP); 2♂, Misiones, Camping del Valle del Cuñá Pirú, Mar 2007, coll. E. Gogliormella (EGO); 1♂, Misiones, 20 Nov 2011, leg. P. Loescher (FCP); 5♂, same locality, 4–7 Nov 2009, leg. L. Aguado (LA); 1♂, same locality, Feb 2013, leg. L. Aguado (LA); 5♂, same locality, 1–3 Jan 2014, leg. L. Aguado (LA); 1♂, same locality, Apr 2014, leg. P. Loescher (FCP); 2♂, same locality, 6 May 2014, leg. L. Aguado, coll. O.J. Espínola (OE); 1♂, Misiones, Dos de Mayo, 6 May 2014, leg. L. Aguado, coll. O.J. Espínola (OE); 2♂, Misiones, Iguazú, 1933, coll. Hayward (MACN); 1♂, Corrientes, “MACN-Bar-Lep-ct 3861” (MACN); 1♂, Formosa, “MACN-Bar-Lep-ct 4028” (MACN); 2♂, Chaco, Charata, 19 Jan 1994, leg. O. Di Iorio (FCP); 1♂, Chaco (FCP); 1♂, same locality, 21 Feb 1980 (FCP); 2♂, same locality, 24 Jan 1981, leg. O. Di Iorio (FCP); 1♂, Tucumán, Depto. Chicligasta, Cochuna, 10 Jan 2011, leg. F.C. Penco (RYB); 1♂, Tucumán, 10 Jun 1968 (FML); 1♂, same locality, coll. Breyer A. (MACN); 2♂, same locality, “Ref. 1848” (MACN); 1♂, same locality, Dec 1946 (FML); 1♂, same locality, ex coll. Weyrauch (FML); 1♂, same locality, 15 Jan 1920 (FML); 1♀, same locality, Jan 1926 (FML); 2♂, same locality, Dec 1925 (FML); 2♂, same locality, Feb 1925 (FML); 1♂, same locality, 26 Oct 1968, leg. Paganini & Barrera (FML); 1♂, Tucumán, Cadillal, Nov 1987, coll. Carpintero (CFA); 1♂, Tucumán, Quebrada de Lules, 25 Jan 1922 (FML); 1♂, Tucumán, Dique Los Pizarros, 10–13 Oct 1982, leg. Golbach (FML); 1♂, Tucumán, Raco, Jan 1939 (FML); 6♂, Tucumán, Taco Ralo, 23 Oct 1968, leg. Paganini & Barrera (FML); 1♂, Tucumán, Concepción, Río Cochuna, Dec 2014, leg. A. Borquez (FCP); 1♂, Córdoba, Dean Funes, 30 Dec 2012, leg. L. Aguado (RYB); 1♂, Córdoba, Huerta Grande, Apr [19]59, leg. H. Förster (RYB); 2♂, 1♀, Córdoba, Alta Gracia, La Granja, Sierras de Córdoba, leg. Bruch (MLP); 1♂, Córdoba (IMZA); 1♂, Córdoba, Villa Dolores (MLP); 1♂, Córdoba, Dean Funes, 30 Dec 2012, leg. L. Aguado (LA); 2♂, 1♀, Córdoba, Calamuchita, El Sauce, leg. Viana (MACN); 1♀, Santa Fe, Depto. Gral. Obligado, Villa Guillermina, 1940, leg. Orfila (FCP); 1♂, La Rioja, coll. Breyer A. (MLP); 1♂, Catamarca, 28 Feb 1993, leg. O. Di Iorio (FCP); 1♀, Entre Ríos, Pronunciamiento, Jan 1972, leg. M. Zelich (FCP); 1♂, Buenos Aires, Saladillo (MACN). **URUGUAY:** 1♂, Montevideo, Feb 1930, det. F. Schweizer (MHNU).

**Diagnosis.** This species is the most easily distinguishable from other *Morpheis* species by external morphological characters, particularly a reduced brownish forewing pattern bounded below by CuA and above by the costa, with borders becoming diffuse toward the apex; a conspicuous wavy pattern interconnecting veins longitudinally; and an elongate uncus in the male genitalia.

**Distribution.** The most widespread species in the genus, ranging from southern Mexico to northern Argentina, excluding the Andean highlands and Caribbean islands (Figure 16B).

**Remarks.** Part of the Herrich-Schäffer collection, including type specimens, was scattered among several European museums [70]; later material from his collection, including types of Cossidae from South America was found in ZISP [71]. Here we designate the male specimen (Figure 14E) as lectotype, and the female (Figure 14I) as paralectotype.

### 3.3. Species Checklist of the Genus Morpheis

1. *Morpheis clenchi* Donahue, 1980

Type locality: USA, Arizona.

2. *Morpheis cognatus* (Walker, 1856)

Type locality: Honduras.

3. *Morpheis comisteus* (Schaus, 1911)

Type locality: Costa Rica.

4. *Morpheis humboldti* Naydenov, Yakovlev & Penco, 2021

Type locality: Brazil, Amazonas.

5. *Morpheis impeditus* (Wallengren, 1860)

Type locality: [unknown].

6. *Morpheis mathani* (Schaus, 1901)

Type locality: Peru, Huambo.

7. *Morpheis melanoleucus* (Burmeister, 1878)

Type locality: Argentina, Buenos Aires.

8. *Morpheis putridus* (Percheron, 1838) **stat. rev.**

Type locality: Brazil.
  *Xyleutes discreta* Dyar, 1940 **syn. nov.**
    Type locality: Brazil.

9. *Morpheis pyracmon* (Cramer, [1780])

Type locality: Suriname.
  *Zeuzera lelex* Dognin, 1891 **syn. nov.**
    Type locality: Venezuela, Merida.

10. *Morpheis votani* (Schaus, 1934)

Type locality: Argentina, La Rioja.

11. *Morpheis xylotribus* (Herrich-Schäffer, [1853])

Type locality: [Brazil].

### 3.4. Key to the Species of Genus Morpheis

**1.** Forewing with brown longitudinal area between CuA and costa; wavy pattern in this area nearly obsolete……………………………………………………………………………***M. xylotribus***
**—** Forewing without brown longitudinal area; area between CuA and costa with same pale ground colour as rest of forewing……………………………………………………………………**2**
**2.** Forewing lacking basal part of dark longitudinal stripe adjacent to costa; in male genitalia, harpe of valve well developed, curved, and sclerotised; lateral processes of juxta with spherical laterally directed broadenings…………………………………………………***M. votani***
**—** Forewing with basal part of dark longitudinal stripe adjacent to costa; in male genitalia, harpe of valve less developed, straight, papilliform; lateral processes of juxta simple, rod-like, without laterally directed broadenings………………………………………………………………**3**
**3.** Fore- and hindwings with wavy pattern reduced to dots or small, disconnected streaks; dark longitudinal stripe on forewing with smooth margins………………………………………**4**
**—** Fore- and hindwings, (especially forewings), with well-developed wavy pattern composed of thin, curved, mostly interconnected streaks………………………………………………………**6**
**4.** Mesonotal area between tegulae dark, strongly contrasting with pale tegulae…………………………………………………………………………………………***M. clenchi***
**—** Mesonotal area between tegulae pale, concolourous with tegulae, with thin dark median longitudinal line…………………………………………………………………………………………**5**
**5.** Male wingspan up to 60 mm; longitudinal stripe on forewing continuous; abdomen dorsally without distinct median dark stripe…………………………………………***M. cognatus***
**—** Male wingspan more than 60 mm; longitudinal stripe on forewing interrupted between proximal costal part and central distal part, or only narrowly connected; abdomen dorsally with distinct median dark stripe…………………………………………………………***M. mathani***
**6.** Fore- and hindwings with grey ground colour, giving specimens darker overall appearance………………………………………………………………………………***M. impeditus***
**—** Fore- and hindwings with pale ground colour…………………………………………………**7**
**7.** Forewing pattern (wavy pattern and longitudinal stripe) light greyish brown, weakly contrasting with ground colour and set off against darker dots along wing outer margin; hindwing with smoky light-grey, weakly expressed wavy pattern……………………………………………………………………………………***M. comisteus***
**—** Forewing pattern (wavy pattern and longitudinal stripe) dark brown, strongly contrasting with ground colour; hindwing with smoky dark pattern or well-developed wavy pattern…………………………………………………………………………………………………**8**
**8.** Fore- and hindwings more rounded, without acute apices; hindwings with distinct smoky dark pattern; imagoes of medium size (male wingspan 41–42 mm) ……………………………………………………………………………………………***M. humboldti***
**—** Fore- and hindwings more elongate, with relatively acute apices; hindwings with well-developed wavy pattern (or only weak smoky dark pattern); imagoes medium-sized to large……………………………………………………………………………………………………**9**
**9.** Median thoracic dorsum dark, sharply contrasting with pale tegulae (in most specimens); forewing with well-developed wavy pattern of thick streaks of uneven width…………………………………………………………………………………***M. melanoleucus***
**—** Median thoracic dorsum entirely pale, or with thin dark median longitudinal line; forewing with wavy pattern of thinner streaks of uniform or uneven width ………………………………………………………………………………***M. putridus* / *M. pyracmon***

### 3.5. Immature Stages of Morpheis

**Figure 18:**
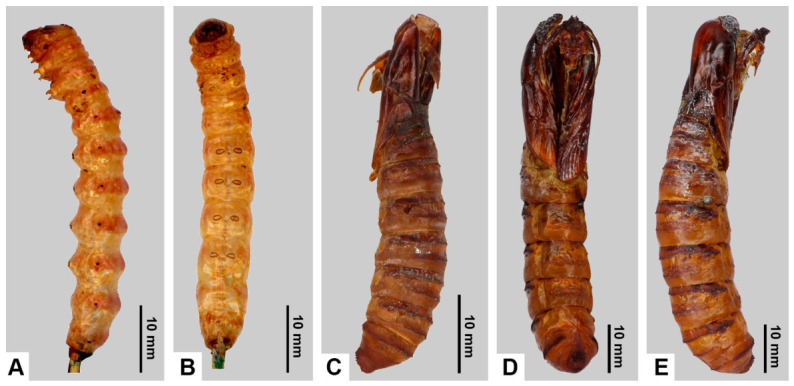
Larva and pupae of *Morpheis* species: (**A**) larva of *M. impeditus*, lateral view; (**B**) larva of *M. impeditus*, ventral view; (**C**) pupa of *M. impeditus*, lateral view; (**D**) pupa of *M. pyracmon*, ventral view; (**E**) pupa of *M. pyracmon*, lateral view.

The only relatively detailed published images of *Morpheis* the caterpillars and pupae are drawings *of M. putridus* [18]. Unfortunately, these images lack a comprehensive description of the preimaginal stages. At the Natural History Museum, London (NHMUK), we discovered and examined dry *M. impeditus* caterpillars from Buenos Aires (erroneously identified as *Xyleutes strigillata*), as well as two dry pupae: one of *M. impeditus* (also misidentified as *X. strigillata*) and one of *M. pyracmon* (Figure 18).

The caterpillar specimen measures 51 mm in length. The body is cylindrical, ochre-coloured. The head capsule, anal shield, and anterior margin of the prothoracic dorsal shield are dark brown, sclerotised; the posterior part of the prothoracic shield bears fine dark tubercles and spines. The head is relatively small, clearly demarcated. The antennae are short, the mandibles are barely noticeable, inconspicuous. Each of the three thoracic segments bears one pair of well-developed, sclerotised, jointed legs; only one pair of spiracles is developed on the thorax. The central part of the abdomen has five pairs of prolegs on segments III–VI. The posterior abdominal segments are damaged, but segment X probably also bears a pair of anal prolegs. Due to the poor condition of the collection specimens, detailed chaetotaxy and setation descriptions are not possible. The pupa of *M. impeditus* measures 46 mm in length, and that of *M. pyracmon* measures 75 mm. The cuticle of dried pupae is dark brown. Head and thoracic appendages are weakly fused to the pupal body. The pupal abdomen is elongate, with well-defined free segments, comprising more than half of its total length. Abdominal segments III–VIII bear two parallel rows of fine spines dorsally; the proximal row is longer than the distal one and extends laterally. Segment IX with one such row, but longer; in *M. pyracmon* specimen it is laterally interrupted. The cremaster is absent, segment X bears a patch of sclerotised spines ventrally.

## 4. Discussion

Despite its relatively large size, wide Neotropical distribution, and economic significance [27,28,29,30], the genus *Morpheis* remains a poorly studied group of Lepidoptera. Characters traditionally used in Lepidoptera taxonomy either exhibit high intraspecific variability (e.g., wing pattern, body size), or, conversely, are interspecifically uniform (e.g., genitalia structure), leading to frequent species misidentifications. The species composition and taxonomic status of many *Morpheis* taxa remain controversial among researchers, and a complete taxonomic revision of the genus is still lacking. Molecular techniques such as DNA barcoding prove powerful tools for studying biodiversity and clarifying phylogenetic relationships in such taxonomically challenging groups, yet they have never been applied to *Morpheis*.

This study presents a comprehensive morphological survey of *Morpheis* and provides the first phylogenetic reconstruction of relationships within the genus, along with molecular species delimitation, based on *COI* sequences. Molecular analyses recover the genus as a strongly supported monophyletic lineage with well-resolved species-level phylogenetic relationships, indicating that the barcode fragment is an informative molecular marker for resolving recent radiations in this group. In contrast, basal branching generally receives less support in both ML and BI analyses.

Our analysis revealed several cases of exceptionally high intraspecific divergence within the genus *Morpheis* (Table S4), specifically in *M. xylotribus* (9.4% ± 1.1%), *M. mathani* (7.1% ± 0.9%), and *M. cognatus* (7.0% ± 1.0%). The observed intraspecific distances are much higher than the ~3% threshold which has been empirically established to define species boundaries in Lepidoptera [72,73]. Although this level is not an absolute threshold for distinguishing species [31], it serves as a useful criterion when assessing the taxonomic status of analysed entities. It should be noted that *Morpheis* taxa showing high intraspecific divergence also split into highly supported clades on the phylogenetic tree, which align well with the geographic distributions of the samples. We assume that such pronounced intraspecific genetic distances found in several *Morpheis* taxa, along with the inferred phylogenetic structure, may reflect overlooked species.

Barcode-based delimitation (ASAP) of 285 specimens also reveals substantially more evolutionary entities (19–21 MOTUs) than the currently recognised species, suggesting considerable cryptic diversity in the genus or strong geographic structuring within some broadly distributed taxa. With the exception of *M. impeditus* and *M. votani*, which form single lineages consistent with current taxonomy, all other commonly recognised species comprise multiple MOTUs (potential species). These results highlight several species complexes as primary targets for further integrative research, requiring denser geographic sampling, deeper morphometric analyses, and expanded multilocus datasets (including nuclear markers) to test whether these lineages represent distinct species or structured populations.

A striking example of discordance between traditional morphology and *COI* lineages is the *M. pyracmon*/*M. putridus* complex. Original illustrations and descriptions indicate *M. putridus* [18] and *M. pyracmon* [3] as morphologically indistinguishable in habitus. In the only study explicitly focused on the genus *Morpheis* [23], *M. putridus* was treated as a junior synonym of *M. pyracmon*. Despite Schoorl’s recognition of *M. putridus* as a valid species [19], specimens exhibiting this habitus have consistently been identified as *M. pyracmon*. Although type material for both taxa is unavailable (presumably lost), their type localities are well documented and geographically distinct: Suriname for *M. pyracmon* and Brazil for *M. putridus*. Our phylogenetic analysis reveals that specimens previously identified as *M. pyracmon* based on external morphology form two distinct, distantly related lineages. These lineages exhibit clear geographic partitioning, with records from Central America and northern South America assigned to one lineage and those from southern South America to the other (Figure 13). The lineages are geographically separated and appear to be allopatric; however, sympatry cannot be ruled out without additional sampling in the potential contact zone. This pattern is congruent with the biogeographic subdivision of the Neotropical Region into the Chacoan and Brazilian subregions [49]. Assuming that the type locality “Brazil” in Percheron’s original description most likely refers to coastal southeastern Brazil, an area that was a key source of collected specimens in the early 19th century, we apply the name *Morpheis putridus* (Percheron, 1838) to the southern lineage and reinstate its species status. Accordingly, we apply the name *Morpheis pyracmon* (Cramer, [1780]) to the northern lineage, whose suggested range includes Suriname. Although these taxa are indistinguishable based on external morphology and genitalia, further detailed morphometric analyses may reveal subtle differences. Moreover, expanded range-wide sampling for DNA barcoding and analysis of additional molecular markers are essential to test and refine the preliminary geographic delimitation of these species.

The systematic revision based on extensive molecular and morphological data presented here also clarifies longstanding uncertainty regarding the taxonomic structure of *Morpheis*. Based on examinations of type specimens, genitalic morphology, and available *COI* barcodes, we delimit the genus to 11 valid species, resolving nomenclatural issues and stabilising *Morpheis* nomenclature. In particular, we reinstate *Morpheis putridus* (Percheron, 1838) (**stat. rev.**) and recognise *Xyleutes discreta* Dyar, 1907 (= *M. discretus* auct.) as its junior synonym (**syn. nov.**). This taxonomic change resolves historical confusion caused by the erroneous interpretation of intraspecific size variation as a species-specific feature and supports the view that size alone is an unreliable taxonomic character in Zeuzerinae; notably, *COI* data confirm conspecificity between small- and medium-sized specimens, unambiguously placing them within the *M. putridus* clade. In parallel, we synonymise *Zeuzera lelex* Dognin, 1891 with *M. pyracmon* (Cramer, [1780]) (**syn. nov.**), based on morphological similarity and congruent distributions with the northern lineage of the synonymised species.

Our phylogenetic results further indicate that informal groupings based solely on the wing pattern can be misleading. Donahue’s “*cognatus* group” (*M. cognatus*, *M. mathani*, *M. clenchi*) [23] recovers as non-monophyletic in our *COI* tree; the presumed diagnostic wing pattern traits appear to have evolved independently in the *M. cognatus* + *M. clenchi* clade and in *M. mathani*. The observed morphological convergence underscores the limitations of external morphology alone for inferring the taxonomic structure of *Morpheis* and highlights the importance of genitalic characters and integrative (morphological and molecular) datasets in Zeuzerinae systematics.

To conclude, our comprehensive taxonomic revision (based on examination of types, updating of species diagnoses, and compilation of biogeographic data), combined with the first molecular assessment of *Morpheis*, establishes a practical baseline for species identification and highlights several species complexes requiring further detailed investigation. Further work integrating multilocus phylogenomic data, detailed morphometric analyses, and improved distributional data for *Morpheis* taxa will be essential to delimit species boundaries, refine species distributions, and resolve deeper relationships within the genus.

## 5. Conclusions

Using an integrative approach based on detailed morphological analysis, examination of type specimens, taxon distributions, and molecular data, we revise the genus *Morpheis* Hübner, [1820] (Lepidoptera: Cossidae), and provide updated diagnoses, comparative illustrations, and verified distribution maps. We clarify several long-standing taxonomic problems and delimit the genus *Morpheis* to 11 valid species. Our results demonstrate the utility of DNA barcoding for resolving species-level phylogenetic relationships in *Morpheis*, a group where traditional characters may show both high intraspecific variability and interspecific uniformity. Phylogenetic analyses confirm the monophyly of the genus and reveal multiple molecular operational taxonomic units (MOTUs) within several morphospecies, suggesting considerable overlooked cryptic diversity in this group.

We conclude that this study establishes a robust taxonomic baseline and provides insights for future integrative research on species boundaries and relationships not only within *Morpheis* but across Zeuzerinae as a whole.

## Data Availability

The original contributions presented in this study are included in the article/Supplementary Material. Further inquiries can be directed to the corresponding authors.

## Supplementary Materials

The following supporting information can be downloaded at: https://www.mdpi.com/article/10.3390/insects17050458/s1, Figure S1: Uncollapsed consensus Bayesian tree of *Morpheis* inferred from *COI* sequences; Table S1: Number of specimens examined for *Morpheis* species; Table S2: Specimens used for molecular phylogenetic analysis; Table S3: Interspecific genetic divergences in *Morpheis*; Table S4: Intraspecific genetic divergences in *Morpheis*.

## Abbreviations

CFA	Colección de la Fundación de Historia Natural Félix de Azara, Universidad Maimónides, Buenos Aires, Argentina.
CMNH	Carnegie Museum of Natural History, Pittsburgh, USA.
CUYO	Ginés Gomariz private collection, Cuyo Region, Argentina.
EGO	Eduardo Gogliormella private collection, Villa Devoto, Buenos Aires, Argentina.
FCP	Fernando César Penco private collection, Morón, Buenos Aires, Argentina.
FML	Fundación Miguel Lillo, San Miguel de Tucumán, Argentina.
IMZA	Instituto de Microbiología y Zoología Agrícola, INTA Castelar, Buenos Aires, Argentina.
LA	Leonardo Aguado private collection, San Fernando, Buenos Aires, Argentina.
MACN	Museo Argentino de Ciencias Naturales “Bernardino Rivadavia”, Buenos Aires, Argentina
MHNU	Museo Nacional de Historia Natural, Montevideo, Uruguay
MLP	Museo de La Plata, Buenos Aires, Argentina
MNHN	Muséum National d’Histoire Naturelle, Paris, France
MWM	Museum Witt Munich, Munich, Germany
NHM-UiO	Natural History Museum, University of Oslo, Norway
NHMUK	The Natural History Museum, London, UK
OE	Oscar José Espínola private collection, Wilde, Buenos Aires, Argentina
OUMNH	Oxford University Museum of Natural History, Oxford, UK
RMNH	Naturalis Biodiversity Center, Leiden, The Netherlands
RYB	Collection of Roman Yakovlev, Barnaul, Russia
USNM	National Museum of Natural History, Smithsonian Institution, Washington, D.C., USA
ZISP	Zoological Institute of the Russian Academy of Sciences, St. Petersburg, Russia
ZSM	Zoologische Staatssammlung München (Bavarian State Collection of Zoology), Munich, Germany
